# Pathways to Care for Critically Ill or Injured Children: A Cohort Study from First Presentation to Healthcare Services through to Admission to Intensive Care or Death

**DOI:** 10.1371/journal.pone.0145473

**Published:** 2016-01-05

**Authors:** Peter Hodkinson, Andrew Argent, Lee Wallis, Steve Reid, Rafael Perera, Sian Harrison, Matthew Thompson, Mike English, Ian Maconochie, Alison Ward

**Affiliations:** 1 Division of Emergency Medicine, University of Cape Town, Cape Town, South Africa; 2 Department of Paediatrics, University of Cape Town, Cape Town, South Africa; 3 Directorate of Primary Health Care, University of Cape Town, Cape Town, South Africa; 4 Nuffield Department of Primary Care Health Sciences, University of Oxford, Oxford, United Kingdom; 5 Department of Family Medicine, University of Washington, Seattle, United States of America; 6 Nuffield Department of Medicine, Oxford University, Oxford, UK and KEMRI-Wellcome Trust Research Programme, Nairobi, Kenya; 7 Paediatric Emergency Medicine, Imperial College, London, UK and NIHR BRC funded researcher Imperial College, London; TNO, NETHERLANDS

## Abstract

**Purpose:**

Critically ill or injured children require prompt identification, rapid referral and quality emergency management. We undertook a study to evaluate the care pathway of critically ill or injured children to identify preventable failures in the care provided.

**Methods:**

A year-long cohort study of critically ill and injured children was performed in Cape Town, South Africa, from first presentation to healthcare services until paediatric intensive care unit (PICU) admission or emergency department death, using expert panel review of medical records and caregiver interview. Main outcomes were expert assessment of overall quality of care; avoidability of severity of illness and PICU admission or death and the identification of modifiable factors.

**Results:**

The study enrolled 282 children, 252 emergency PICU admissions, and 30 deaths. Global quality of care was graded good in 10% of cases, with half having at least one major impact modifiable factor. Key modifiable factors related to access to care and identification of the critically ill, assessment of severity, inadequate resuscitation, and delays in decision making and referral. Children were transferred with median time from first presentation to PICU admission of 12.3 hours. There was potentially avoidable severity of illness in 185 (74%) of children, and death prior to PICU admission was avoidable in 17/30 (56.7%) of children.

**Conclusions:**

The study presents a novel methodology, examining quality of care across an entire system, and highlighting the complexity of the pathway and the modifiable events amenable to interventions, that could reduce mortality and morbidity, and optimize utilization of scarce critical care resources; as well as demonstrating the importance of continuity and quality of care.

## Introduction

The ideal healthcare system would identify sick children early in their illness, and provide treatment that is safe, effective, patient centred, efficient, timely and equitable [1]. To focus quality improvement, we need to understand the current situation: the processes, time taken, and the nature and quality of care provided during each step. Quality of care and speed through health systems are related to the variety of conditions encountered [2], thus provision of optimal care to all children with a wide range of life threatening illness or injury may be extremely challenging [3]. Highlighting foci for effective quality improvement interventions in paediatric emergencies has the potential to provide significant benefit, especially in resource poor settings where the need is greatest, and the health services poorest [4], but also in more affluent countries where delivery of recommended care may still be very difficult [5]. The fundamental challenge of a “pathway to care” is that optimal results can only be expected if the entire process functions effectively.

We could find no studies in lower resource settings assessing the quality of care delivered to critically ill or injured children from the time of first presentation through to death or admission to intensive care. In the United Kingdom (UK), the Confidential Enquiry into Maternal and Child Health (CEMACH) has been in effect for more than 50 years, now including assessment of childhood mortality and morbidity [6–8]. There have been improvements in healthcare for mothers and children in the UK, which many attribute substantially to the CEMACH findings and recommendations [8–11], and widespread use of this process in other settings [12–18], such that it is now widely regarded as a framework for mortality review.

Although South Africa is considered a middle income country, and the Western Cape one of the wealthier provinces, there is much inequity in socio-economic and health status [19], with a third of the population living below international poverty lines, and a quarter of children living in informal and overcrowded houses, much as in many developing countries [20]. South Africa’s health system is divided, with the state funded public sector providing relatively resource constrained care to more than two thirds of the low income population, and a private sector, funded predominantly by medical insurance, serving the wealthier and formally employed population.

We undertook a study to identify preventable failures in the care of critically ill or injured children and thus identify key areas for interventions to optimise care. The objectives were to: i) describe the details of the entire care pathway to a paediatric intensive care unit (PICU) or death, ii) use expert clinical review to evaluate the quality of care along the pathway and identify preventable failures in the care provided.

## Materials and Methods

### Study Design

The study was conducted at the Red Cross War Memorial Children’s Hospital (RCWMCH), Cape Town, one of only two public tertiary hospitals managing children from throughout the Western Cape Province. Tygerberg Hospital in Cape Town has a smaller PICU, generally limited to medical patients (predominantly neonates) and post-surgical patients (elective), with a different referral area from RCWMCH although there is limited overlap. Referrals are primarily from the surrounding metropolitan area, with a paediatric population of 930 000 [20] by: approximately 600 independent general practitioners; 109 nurse-led clinics (Clinics); 45 doctor-led primary health centers (36 operate only in office hours; 9 are 24 hour) (Community Health Centers (CHC)); and 7 hospitals. Patients are transferred by the Emergency Medical Services (EMS) with 140 ambulances across the province.

The study was approved by the Faculty of Health Sciences Research Ethics Committee, University of Cape Town (UCT HREC 211/2011); and the Oxford Tropical Research Ethics Committee, Oxford University (OXTREC 29–11), as well as the Western Cape Department of Health and the City of Cape Town. The study was performed in accordance with the ethical standards laid down in the 1964 Declaration of Helsinki and its later amendments. As per ethics approval, written informed consent for caregiver interview, and collection and analysis of the child's medical records was obtained from the parent or caregiver of every enrolled child in the study. The signed paper consent forms were securely stored with the study records. Patient identity was encrypted by the database, and paper records securely stored to retain anonymity and confidentiality.

**Fig 1:**
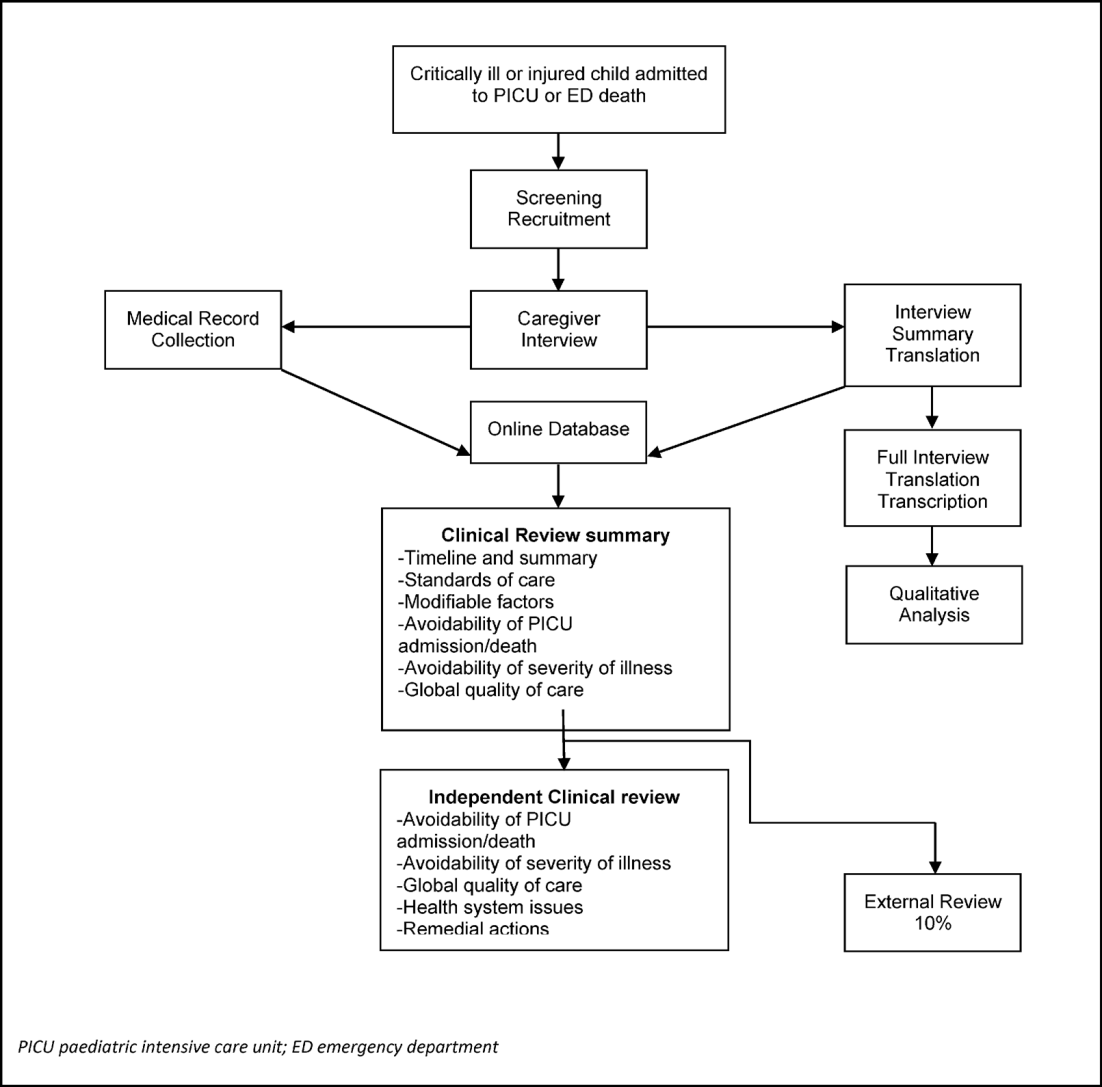
Flow Chart of Pathways to Care research project recruitment, data collection and clinical review process.

We recruited critically ill children on admission to PICU, or when they died in an emergency department (ED) (**Fig 1**). The inclusion criteria were: aged <13 years, emergency PICU admission during recruitment phases (alternate weeks) over a year; death in the RCWMCH-ED; or death in the ED of the immediate geographic referring facilities. Exclusion criteria were: elective PICU admission; inpatient for >5 days prior to PICU admission; children under palliative care, or those dead on arrival to the ED.

Following written informed consent, the caregiver of each child was interviewed, focusing on the caregiver’s narrative of the healthcare episode, access, timeline, and perceptions of care. Medical records were obtained from each healthcare facility and EMS transfer involved. Clinical outcomes collected for each child were the Paediatric Index of Mortality (PIM2) [21] score on admission to PICU; duration of stay in PICU and in RCWMCH; and the 30 day outcome.

### Clinical Review

We developed a method of expert clinical review modelled on the CEMACH [6, 7]. A list of potential modifiable factors was compiled (**S1 Table**) based on those identified in the CEMACH, and the Child Healthcare Problem Identification Programme (CHIP) in South Africa [22]. We categorised the possible impact of these factors:

**Major**: factor which had clear negative impact on the outcome for the patient and worsened mortality or morbidity; directly and overwhelmingly important factor in the severity of illness/death
**Moderate**: factor which on its own had minimal negative impact on the outcome but may have caused some morbidity and/or extended the hospital/PICU stay
**Near Miss**: unplanned event that did not have major impact–but had the potential to do so—only a fortunate break in the chain of events prevented an injury, fatality or damage
**No Defined Impact**: factor which has no individual or cumulative negative impact on the outcome of this or future cases
**Not known**: cannot be established or estimated given facts known about scenario

Standards of care were developed for paediatric emergency care and referrals for facilities and EMS transfers using a modified Delphi technique with stakeholders (representing each healthcare level) prior to the study onset as there were no locally agreed care standards—these were categorised into critical, important and necessary (**S2 Table**). We also considered whether the health care prior to the acute episode was appropriate and if better care could have prevented the severity of illness or outcome.

One clinician (PH) summarized each case, developing a timeline of the pathway based on both the medical record and interview data. Three senior local clinicians, all leading clinicians and researchers in their fields, one each from paediatric intensive care (AA), emergency medicine (LW), and primary care (SR) then independently reviewed each case using all available information.

### Outcomes for each child:

1. Overall global quality of care for the entire pathway
2. Avoidability of death
3. Avoidability of PICU admission
4. Avoidability of severity of illness on PICU admission
5. Number and type of modifiable factors
6. Presence of healthcare system issues

### Outcomes for each healthcare contact/ transfer:

7 Number and type of modifiable factors
8 Compliance with consensus standards of care for facility level/ transfer

Conflicting assessments on outcomes 1 to 4 were resolved by discussion. A UK based paediatric emergency medicine specialist (IM) undertook external review of a random 10% of cases.

### Sample Size

Pilot data suggested that the recruitment of 470 children would be possible, giving a precision of 4% based on a 95%CI on a proportion of 40% of children suffering from modifiable factors in their care. For our sample size calculation we did not assume a finite population. The estimate of the SE for the proportion is therefore SE(p) = sqrt ((p x (1-p) / n); where n is the required sample size. The precision defined is equal to 1.96 x SE(p)—that is half of the 95% Confidence Interval. A sample of 470 participants therefore would have provided a precision of 4.43% for a proportion of 40%. The expected annual emergency admissions to the RCWMCH ED for the study period were approximately 55 000. Correcting for this population size, a sample of 466 would have provided us with the same precision of 4.43% for the same proportion (40%).

### Data Analysis

For demographics, diagnosis, pathway variables, and modifiable factors, we used descriptive statistics (median and interquartile ranges (IQR), or mean and standard deviation (where normally distributed) for continuous variables, and n(%) for the categorical variables). To assess agreement between each of the reviewers’ blinded assessments (of outcomes 1–4) and the final consensus assessment we estimated kappa statistics [23]. Analyses were performed using SPSS [24].

## Results

**Fig 2:**
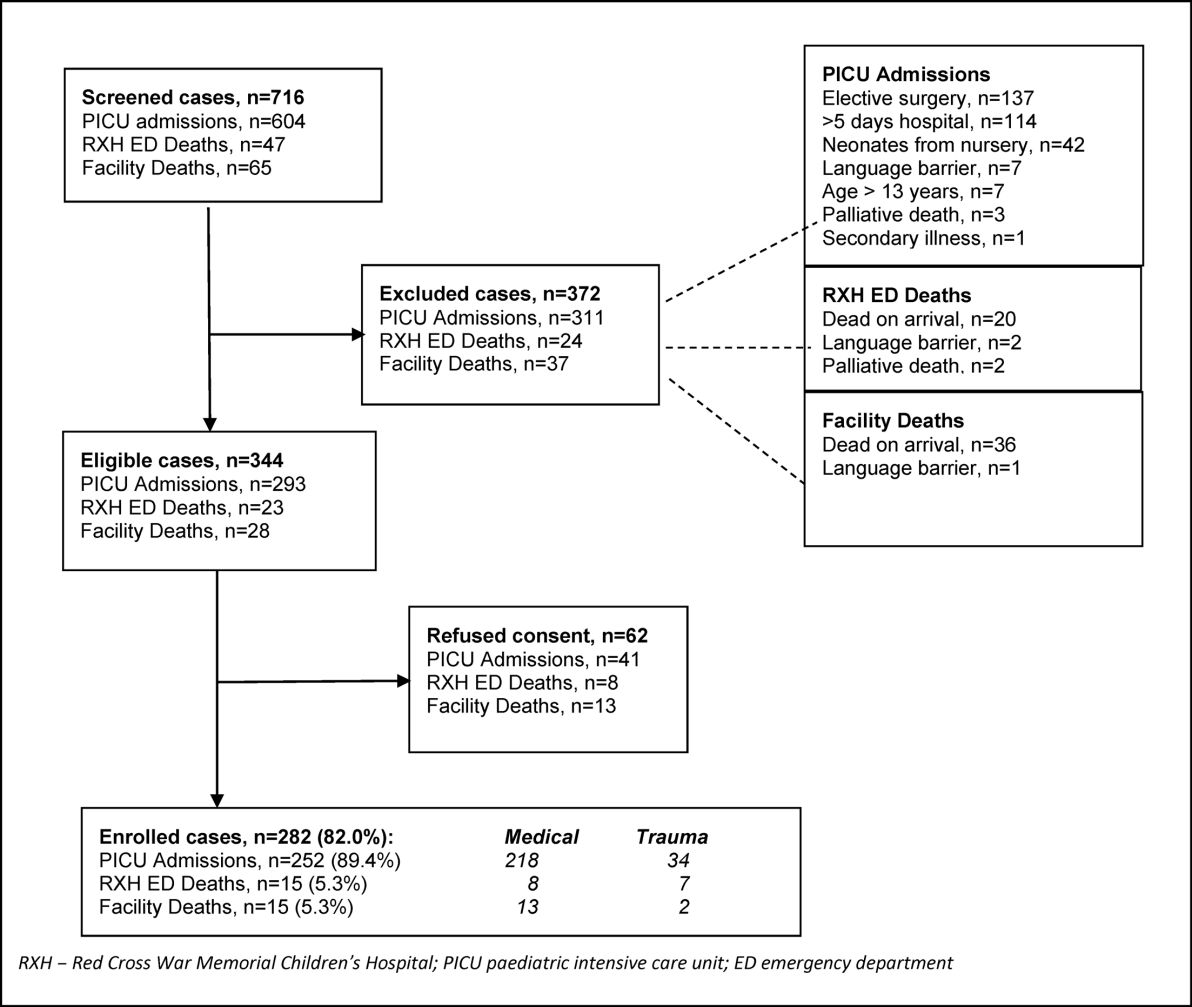
Recruitment process and outcomes.

From 1st November 2011 to 31st October 2012, 716 cases were screened, of which 344 were eligible, and 282 (82.0%) agreed to participate (**Fig 2**). A total of 62 eligible caregivers (18.0%) refused to participate. There was a higher refusal rate in those caregivers whose children had already died at the time of recruitment (21/51, 41.2%). Most enrolled children were admitted to the PICU (252, 89.4%), with 15 deaths in RCWMCH-ED (5.3%) and 15 deaths at other EDs (5.3%). The majority of children (239, 84.8%) had medical illnesses; the rest were related to trauma.

### Participant Characteristics

Over half the children were male (164, 58.2%); 162/239 (67.8%) medical cases were predominantly under a year of age whereas 22/43 (51.2%) of trauma cases were older than 5 years (**Table 1**). The primary diagnoses of the 239 medical cases were respiratory (102, 42.7%) (mainly infection), followed by sepsis (42, 17.6%), and 97 (42.7%) of the medical cases were underweight for age (z<-2). Comparison of the cohort with the provincial census data [20] is shown in **S3 Table.** Data from the interviews showed less than a third of households (85, 31.1%) had an income over $300 per month. Transport to the nearest healthcare facility was on foot for 199 (70.5%) cases and to the nearest 24 hour facility (median 6.0 km (IQR 2.5–13.0) from home) via public transport for 154 (54.6%). Most children (254, 90.1%) lived within the Cape Town metropol.

**Table 1:** Demographics, acute referral timeline and clinical outcomes for the cohort of children.

DEMOGRAPHICS	Medical (n = 239)	Trauma (n = 43)	Total (n = 282)
Gender: Male	137(57.3%)	27(62.8%)	164(58.2%)
Age of Child Median (months) (IQR)	4.8(2.2–20.2)	63.5(23.5–63.5)	7.8(2.5–33.6)
<1 month	30(12 6%)	0(0 0%)	30(10 6%)
1 month to 1 year	132(55 2%)	5(11 6%)	137(48 6%)
1 year to 5 years	55(23 0%)	16(37 2%)	71(25 2%)
>5 years	22(9 2%)	22(51 2%)	44(15 6%)
**Distance from Health Facility**			
Nearest facility (km) Median(IQR)	2 0(0 8–4 0)	1 0(0 5–2 0)	2 0(0 5–3 0)
Nearest 24 hour facility (km) Median(IQR)	6 0(3 0–12 0)	7 0(2 0–15 0)	6 0(2 5–13 0)
**Diagnosis**			
Trauma^a^	-	43(100.0%)	43(15.3%)
cardiac^b^	30(12.6%)	-	30(10.6%)
gastroenteritis	13(5.4%)	-	13(4.6%)
neurological-meningitis/epilepsy^c^	20(8.4%)	-	20(7.1%)
respiratory disease^d^	102(42.7%)	-	102(36.2%)
sepsis/ septic shock^e^	42(17.6%)	-	42(14.9%)
other^f^	32(13.4%)	-	32(11.3%)
**Expected weight for age (z-score)**^g^	**(n = 227)**	**(n = 32)**	**(n = 259)**
z < -3	67(29 5%)	0(0 0%)	67(25 9%)
-3 < z < -2	30(13 2%)	0(0 0%)	30(11 6%)
z > -2	130(57 3%)	32(100%)	162(62 5%)
**Length of pathway all children (Median (IQR))**	**(n = 239)**	**(n = 43)**	**(n = 282)**
Onset of illness to first presentation (days)	2 (0–3.0)	0 (0.0–0.0)	1 (0.0–3.0)
First presentation to RCWMCH arrival (hours)	4.4 (1.9–9.2)	1.9 (1.0–5.2)	4.2 (1.7–8.9)
First presentation to PICU admission (hours)	13.8 (7.3–46.0)	9.8 (6.3–16.0)	12.3 (6.9–39.6)
RCWMCH arrival to PICU admission (hours)^h^	5.0 (2.4–15.9)	5.5 (3.1–8.1)	5.0 (2.5–12.9)
EMS activation to destination facility (minutes)^i^	**(n = 237)**	**(n = 55)**	**(n = 292)**
	86.0 (56.0–124.0)	80.0 (48.0–128.0)	86.0 (54.0–124.0)
**Clinical Outcomes for PICU Admissions only**	**Medical (n = 218)**	**Trauma (n = 34)**	**Total (n = 252)**
**Outcome at 30 days**			
Death in/after PICU	26(11.9%)	2(5.9%)	28(11.1%)
Discharge home	150(68.8%)	13(38.2%)	163(64.7%)
Remain inpatient	42(19.3%)	19(55.9%)	61(24.2%)
	*Median (IQR)*	*Median (IQR)*	*Median (IQR)*
**Risk of mortality**^j^ **(PIM2%)**	6.9(1.8–18.2)	7.6(4.6–12.6)	6.9(2.0–16.6)
**PICU Length of stay (hours)**	73.6 (43.0–159.4)	94.5 (43.6–218.7)	76.9 (43.0–164.0)
**Total RCWMCH Length of stay (days)**	10.5(7.0–20.0)	15.0(9.8–25.8)	11.0 (7.0–21.0)

IQR inter quartile range; RCWMCH Red Cross War Memorial Children’s Hospital; PICU paediatric intensive care unit; EMS emergency medical services

^a^ trauma: road traffic accidents(28), burns (8) and other (7) non road traffic accident injury

^b^ cardiac: congenital heart disease (17) and myocarditis/ cardiomyopathy (13)

^c^ neurology includes meningitis (14), epilepsy(3);

^d^ respiratory: infective (pneumonia/bronchiolitis) (82); obstructive airway/croup/asthma (13)

^e^ sepsis/ septic shock: neonatal (18), older infants/ children (24)

^f^ other includes: surgical (12),death unknown causes (7), overdose (3), drowning (2), renal failure, diabetic keto-acidosis, hepatic failure

^g^ z-score—WHO Global Database on Child Growth and Malnutrition (data incomplete–no age/ weight z score for > 10 year olds)

^h^ 32 patients went directly to PICU on arrival at RCWMCH (all had been previously accepted by PICU with a bed reserved for them); medical(31), trauma (1)

^i^ EMS was not utilized by all cases but some cases had more than one EMS transfer

^j^ on admission to PICU PIM2 score–Paediatric Index of Mortality [21]

### Pathway Characteristics

**Fig 3:**
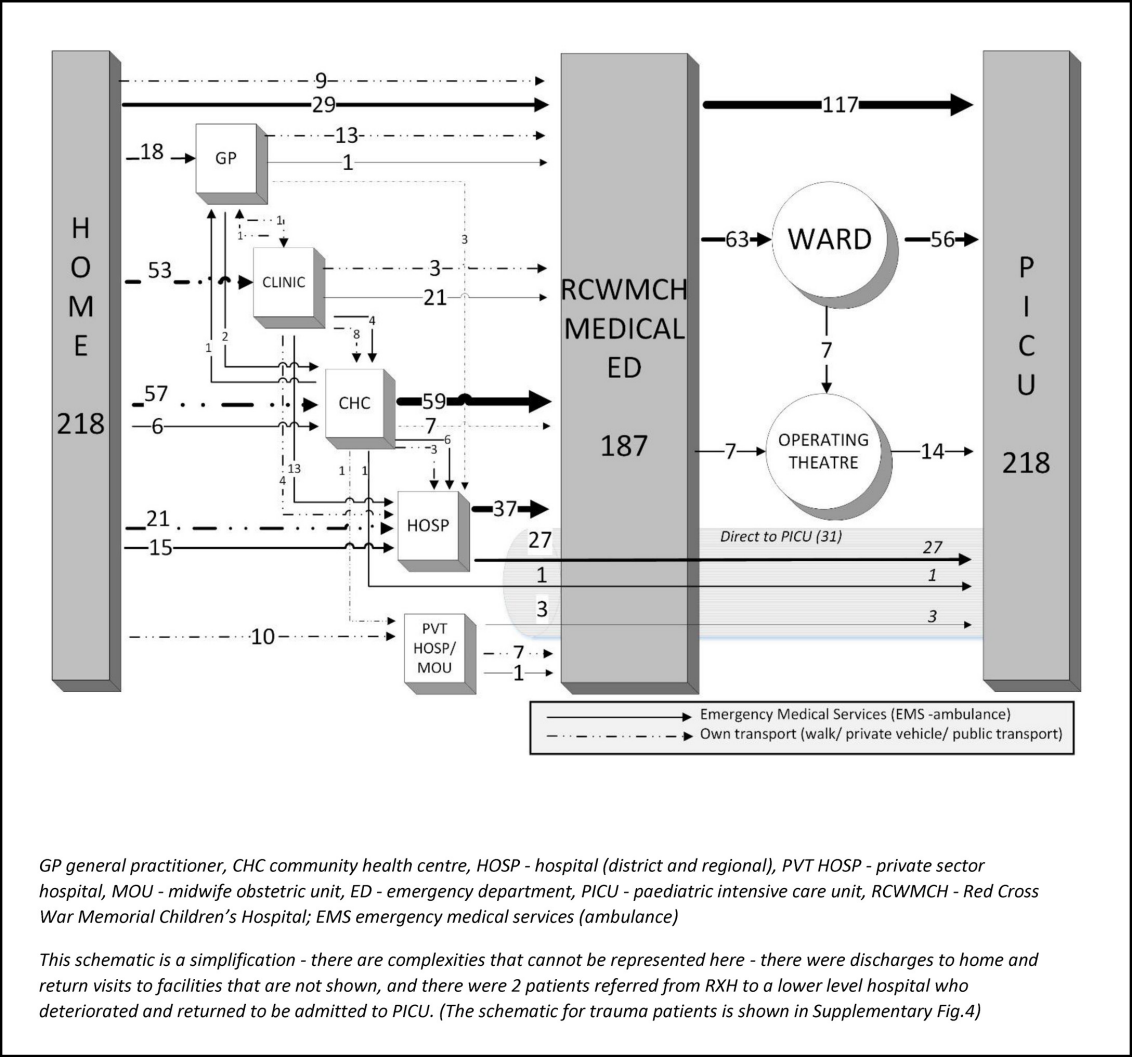
Schematic of the referral pathway for medical patients admitted to Paediatric Intensive Care.

**Fig 4:**
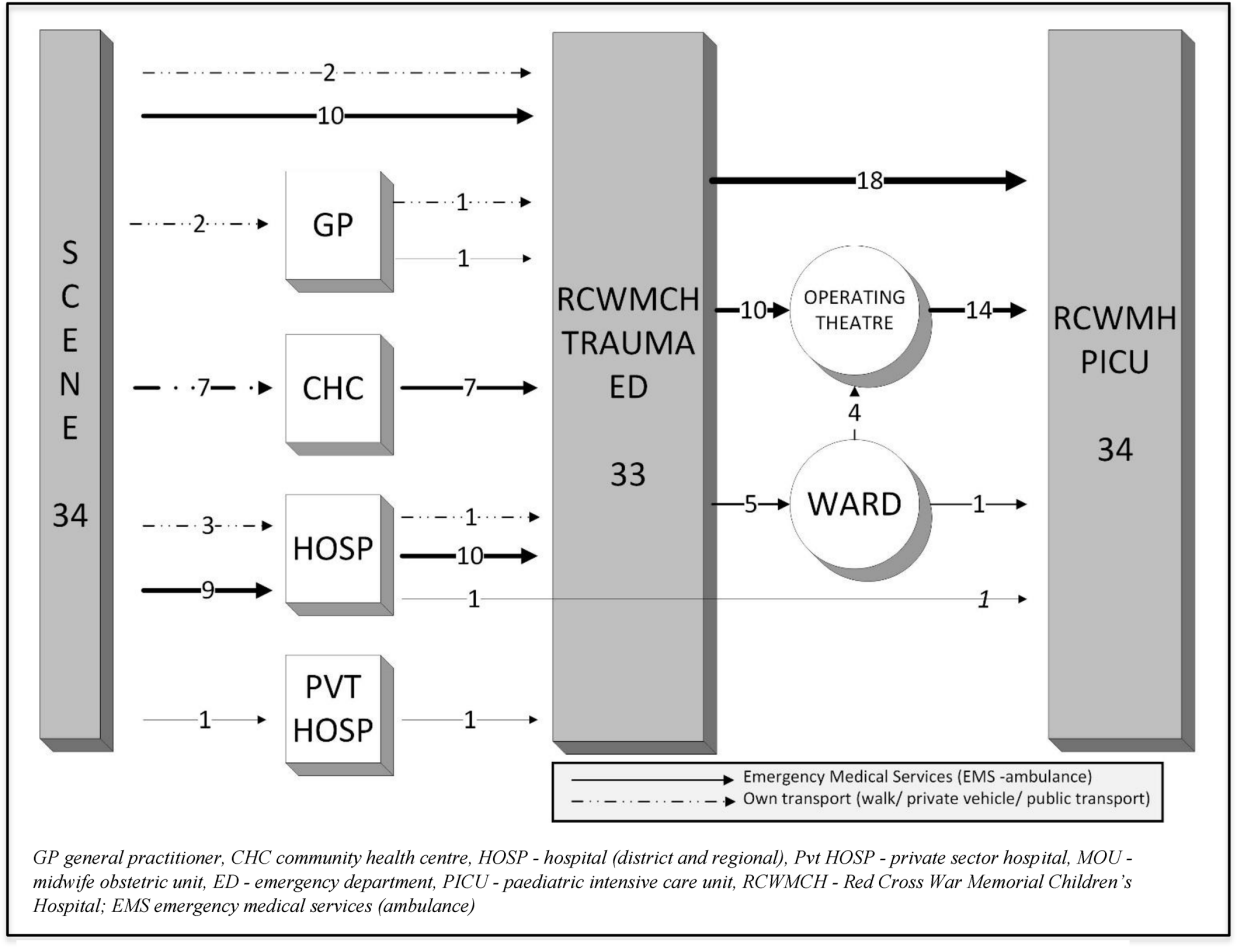
Schematic of the referral pathway for trauma patients admitted to Paediatric Intensive Care.

Over the study period data were collected from 641 consultations across 22 GPs, 32 Clinics, 6 CHCs, 20 Regional/District hospitals, 292 EMS transfers, and from RCWMCH. A fifth (52/239, 21.8%) of caregivers of children with medical problems had consulted elsewhere prior to their first presentation at a healthcare facility and only a third (92/282, 32.6%) of caregivers knew the number to call for EMS. Most children (140/239, 58.6%) with medical illness accessed a primary healthcare facility (GP, Clinic or CHC), while trauma cases were more often seen initially by EMS (25/43, 58.1%) rather than by primary health care (12/43, 27.9%). The majority presented outside working hours (148/239, 61.9% medical cases; 34/43, 79.1% trauma cases). Prior to arrival at RCWMCH, 170/282 (60.3%) children were seen at just a single facility. Children’s pathways from home through healthcare facilities to the PICU were often highly complex, particularly for medical cases (**Figs 3 and 4)**.

### Timing of transitions through the pathway

All children with trauma presented to RCWMCH on the day of injury, whilst 76.6% (183/239) of medical cases presented within 3 days of illness onset (**Table 1**). Overall, 179 (75%) of medical patients arrived at RCWMCH within 9.2 hours of presenting to the first facility, and for trauma (32) (75%) of trauma patients within 5.2 hours. For those children admitted to PICU, 135/252 (53.6%) were admitted directly from the ED in a median of 4.1 hours (IQR 2.7–5.8). Other children were admitted to wards and had a longer and often overnight stay prior to PICU admission (**S4 Table**).

### Child Based Outcomes

On admission to PICU, the median predicted risk of mortality (PIM2) for the medical cases was 6.9%, and 7.6% for trauma (**Table 1)**. Total RCWMCH length of stay was longer for the trauma cases (median 15.0 days, IQR 9.8–25.8) than the medical cases (10.5 days, IQR 7.0–20.0). By day 30, almost two-thirds of the children had been discharged (163/252, 64.7%), a quarter remained in hospital (61/252, 24.2%) and the remainder had died (28/252, 11.1%).

The global quality of care was graded good in 29 (10.3%) of all cases, with the majority graded as fair (196, 69.5%) (**Table 2)**. For the 30 children who died prior to PICU admission, death was considered avoidable or potentially avoidable in more than half (17, 56.7%). For the 252 admitted to PICU, admission was considered avoidable or potentially avoidable for 61 (24.2%), and the severity of illness/injury on admission avoidable or potentially avoidable in 185 (73.4%). Three quarters of cases (210, 74.5%) had clear or likely health system issues. A total of 3212 modifiable factors were identified for the 282 children, with half (141, 50.0%) having at least one major impact modifiable factor, whilst the majority (267, 94.7%) had at least one moderate impact factor. The two most frequent major impact modifiable factors associated with poor outcomes (i.e. poor global quality of care and avoidable: severity of illness, PICU admission and death) (**S5 Table**) were: inadequate initial assessment/interpretation of severity, and resuscitation not done or inadequate for a shocked patient. Others associated with poor outcomes were: delays in critical management decisions, inadequate access to emergency care or personnel, and referral delays.

**Table 2:** Outcomes of Expert Review.

	Medical	Trauma	Total
**Global Quality of Care**^a^	(n = 239)	(n = 43)	(n = 282)
Poor	55(23.0%)	2(4.7%)	57(20.2%)
Fair	166(69.5%)	30(69.8%)	196(69.5%)
Good	18(7.5%)	11(25.6%)	29(10.3%)
**Avoidability of Death**	(n = 21)	(n = 9)	(n = 30)
Not Avoidable	6(28.6%)	7(77.8%)	13(43.3%)
Potentially Avoidable	12(57.1%)	2(22.2%)	14(46.7%)
Avoidable	3(14.3%)	0(0.0%)	3(10.0%)
**Avoidability of PICU**	(n = 218)	(n = 34)	(n = 252)
Not Avoidable	161(73.9%)	30(88.2%)	191(75.8%)
Potentially Avoidable	52(23.9%)	4(11.8%)	56(22.2%)
Avoidable	5(2.3%)	0(0.0%)	5(2.0%)
**Avoidability of Severity**	(n = 218)	(n = 34)	(n = 252)
Not Avoidable	49(22.5%)	18(52.9%)	67(26.6%)
Potentially Avoidable	155(71.1%)	15(44.1%)	170(67.5%)
Avoidable	14(6.4%)	1(2.9%)	15(6.0%)
**System Issues**^b^	(n = 239)	(n = 43)	(n = 282)
No	36(15.1%)	36(83.7%)	72(25.5%)
Possibly	72(30.1%)	3(7.0%)	75(26.6%)
Yes	131(54.8%)	4(9.3%)	135(47.9%)
**Number of Modifiable Factors**^c^ **median (range; IQR)**			
Major Impact	1 (0–16; 0–3)	0 (0–8; 0–1)	1 (0–16; 0–3)
Moderate Impact	6 (0–19; 4–10)	5 (0–13; 3–8)	6 (0–19; 3–9)

PICU paediatric intensive care unit; IQR inter quartile range

^a^ grading of quality of care was performed relative to the expectations of reviewers: poor—health care which was clearly below the average expectations of the facility/health care provider (HCP); fair–health care of an average level expected of the facility/HCP; good–health care at an excellent level above average expectations

^b^ System Issues—defined as potential healthcare interventions prior to the acute episode which could have had a positive impact on the health of the child prior to the acute critical illness. (e.g. missing long term deterioration at a prior consultation or inadequate follow up of a high risk baby)

^c^ grading of Modifiable Factors: major (clear negative impact on the outcome for the patient), moderate (minimal negative impact on the outcome but likely caused some morbidity and/or extended the illness duration) (e.g. failure to administer a fluid bolus in a shocked child would be a major MF, delay in administration of antibiotics to a child with respiratory distress (of unclear aetiology) a moderate MF)

(a total of 3212 modifiable factors were identified for the entire cohort (comprising 477 (14.95) major, 1826 (56.9%) moderate, 44 (1.4%) near miss, 290 (9.0%) no defined impact and 575 (17.9%) unknown impact modifiable factors)

#### Facility Based Outcomes

The five most frequent major and medium impact factors for consultations at the main facilities are presented in Table 3. At both the clinics and 24hr CHCs, there were four common major impact modifiable factors: inadequate access to emergency care or personnel, inadequate assessment/interpretation of severity, resuscitation not done or inadequate and management of shock/ circulatory collapse. A similar pattern was noted in hospital EDs, where the four common major impact modifiable factors were: inadequate assessment/interpretation of severity, resuscitation not done or inadequate, delays in critical management decisions and issues with antibiotic therapy (additional details in **S6–S8 Tables**).

**Table 3:** Five most frequently identified major and moderate impact modifiable factors for the main facilities and for EMS transfers.

Major Modifiable Factors^a^	N(%^b^)	Moderate Modifiable Factors^a^	N(%^b^)
**City Health Clinic (n = 57 consultations)**			
Accessibility of Emergency Care area/ personnel	12(21.1)	Accessibility of Emergency Care area/ personnel	15(26.3)
Inadequate assessment at triage	10(17.5)	Inadequate assessment at triage	11(19.3)
Inadequate assessment/ interpretation of severity	8(14.0)	Antibiotic therapy	11(19.3)
Resuscitation not done/ inadequate for shocked patient	7(12.3)	Ventilatory Management	9(15.8)
Circulatory management	4(7.0)	Explanation to caregiver	9(15.8)
**24 Hour CHC (n = 103 consultations)**			
Resuscitation not done/ inadequate for shocked patient	25(24.3)	Antibiotic therapy	25(24.3)
Inadequate assessment/ interpretation of severity	17(16.5)	Inadequate assessment/ interpretation of severity	24(23.3)
Circulatory management	14(13.6)	Explanation to caregiver	24(23.3)
Accessibility of Emergency Care area/ personnel	10(9.7)	Accessibility of Emergency Care area/ personnel	22(21.4)
Missing key findings (history/ clinical)	6(5.8)	Ongoing monitoring/management while awaiting transfer	19(18.4)
**Hospital (District & Regional) (n = 95)**			
Inadequate assessment/ interpretation of severity	8(8.4)	Ongoing monitoring/management while awaiting transfer	20(21.1)
Resuscitation not done/inadequate for shocked patient	7(7.4)	Delay in disposal decisions	20(21.1)
Accessibility of emergency care area/ personnel	5(5.3)	Antibiotic therapy	14(14.7)
Delay in critical management decisions	5(5.3)	Accessibility of Emergency Care area/ personnel	12(12.6)
Antibiotic therapy	4(4.2)	Delay in critical management decisions	12(12.6)
**RCWMCH ED (n = 241 consultations)**			
Referral Delay	11(4.6)	Ongoing monitoring/management while awaiting transfer	106(44.0)
Resuscitation not done/inadequate for shocked patient	11(4.6)	Referral Delay	97(40.2)
Inadequate assessment/ interpretation of severity	9(3.7)	Antibiotic therapy	51(21.2)
Delay in critical management decisions	7(2.9)	Delay in critical management decisions	47(19.5)
Antibiotic therapy	4(1.7)	Delay in disposal decisions	34(14.1)
**EMS (n = 292 transfers)**			
Inappropriate vehicle/ crew/ equipment	20(6.8%)	Explanation to caregiver	67(22.9%)
Response time delay	19(6.5%)	Inappropriate vehicle/ crew/ equipment	65(22.3%)
Inadequate stabilization for transfer	10(3.4%)	Inadequate monitoring en route	44(15.1%)
Inadequate assessment before transfer	6(2.1%)	Response time delay	43(14.7%)
Dispatch time delay	6(2.1%)	Inadequate assessment before transfer	43(14.7%)

CHC community health centre; RCWMCH Red Cross War Memorial Children’s Hospital; ED Emergency Department; EMS emergency medical services

^**a**^Modifiable Factor Impact: Major–factor which had clear negative impact on the outcome for the patient (worsened mortality or morbidity)—a directly and overwhelmingly important factor in the severity of illness/ death; Moderate–factor which on its own had minimal negative impact on the outcome but may have caused some morbidity and/ or extended the hospital/ PICU stay

^b^ Percent of modifiable factor per consultations/ transfers at each facility/ EMS transfer

Agreement between the internal clinical reviewers and the consensus (after discussion) for the four main outcomes was moderate to substantial (kappa ranged from 0.471–0.864). The external reviewer generally rated care as better than the internal reviewers, with lower agreement (kappa ranged from 0.339–0.458; not estimable for avoidability of death (small numbers) and negative for global quality of care).

Each of the applicable standards **(S2 Table)** were applied to each facility visit and EMS transfer. A variable number of standards were applied to each site/transfer dependent on the context, diagnosis and information available. The rate of compliance with all standards, increased from primary health care sites (23.5% for GP, and 50.9% for clinics) through to hospital based sites (74.4% for district hospitals and 77.5% at RCWMCH ED) (**S9 Table)**. Compliance with standards was higher for all types of EMS contacts (82.3% overall).

## Discussion

This study evaluated the nature and the quality of care for critically ill or injured children within the setting of a metropolitan health service of a middle income country which is relevant to many other settings globally. Overall quality of healthcare was good in only a small minority of children; our findings demonstrate that the pathway to care from initial healthcare contact in community settings to PICU admission or death is complex and has considerable scope for improvement. The most frequent clinical failings were in the areas of assessment and initial resuscitation, compounded by delays in decision making and referral.

The methodology was based on the CEMACH approach, however, there are a number of unique features. This is the first study to review clinical care of a large number of critically ill children (with a wide range of diagnoses) in a resource constrained setting, with high enrolment rates and using a detailed review process, with the perspective of the family from caregiver interviews. The methodology in this study has provided information on multiple aspects of quality of care and has highlighted the complexity of quality analysis in a healthcare system which will have relevance to healthcare systems at all levels of resource availability.

Families of patients admitted to the study were poorer, more likely to be unemployed and to live in informal dwellings when compared to the provincial census data (**S3 Table**) [20]. This may have contributed to the difficulties in accessing healthcare as reflected by multiple visits to healthcare facilities prior to the onset of the life-threatening episode, delays in seeking help, and underutilization of emergency services.

Given that most families did not have access to private transport, the distance to after-hours healthcare facilities (the commonest site of acute presentations) is a significant challenge for improvement. Although individual facilities may be able to upscale after-hours resources, a system wide approach including empowering and educating parents about when, where and how to access care is indicated. Improving the ways parents can communicate concerns about acutely unwell children; alerting parents of high risk neonates on worrying signs and emergency access are two examples.

The high frequency of specific clinical failings in the pathway suggests further areas for interventions. Particular diagnoses such as respiratory tract infections were both common and inadequately managed, suggesting an educational intervention could be focussed on a relatively small group of conditions. The CEMACH enquiry [15] highlighted that failure, to recognise and manage serious infection was the most frequent avoidable factor in primary care. While poor recognition of illness severity in infants and small children is a common problem in EDs and district or regional hospitals [25], data from the primary care setting has highlighted the non-specificity of clinical signs in the early phases of severe sepsis [26]. Studies of children with meningococcemia [25, 27] and severe bacterial infection [28] in well-resourced settings, have identified recognition and management of seriously ill children as key issues. Education and training could focus on recognition of acutely unwell children, including recognition of ‘red flag’ features, measurement of vital signs and accurate triage scoring, and the value of parental and clinician ‘gut feeling’ (including the potential significance of repeat visits).

A body of evidence in hospitalised children in high resource settings has demonstrated the effectiveness of early warning systems for recognition of critically ill and deteriorating children [29–32]. Thus investment in quality of care improvement in CHCs and EDs (at all levels), in keeping with data from Kenya [33–36], would appear to be potentially more effective in improving outcomes, particularly as these centres saw many ill children.

A major concern is the duration of the pathway to PICU admission, which were consistently unacceptably long [37]. Pathways were complex, on occasion reflecting developing illness, but omission of certain steps could have substantially reduced the time taken. Referral delays could be reduced by optimizing system efficiencies, such as fast tracking patients directly to PICU, better prioritization of EMS services, and early warning systems in the hospital setting. A centralized paediatric emergency line, could be a useful avenue for emergency advice, as well as facilitating decision making and co-ordination of appropriate transfer and referral destination. The longest delays were in the EDs of the tertiary referral hospital, often a relating to access to the intensive care unit.

The study had some limitations. We enrolled a smaller sample of children than anticipated due to stringent exclusion criteria but since the proportion of children with good clinical care (10%), was lower than originally expected, recruitment of 282 children provided a precision of 4% (95% CI 7%-14%) consistent with what was felt to be clinically meaningful during study design. Reviewers were not blinded to the case outcome which may have introduced a bias when reviewing care, but we attempted to reduce this bias by clear, written definitions of categories, multiple reviews and consensus building. Agreement between different reviewers was initially moderate, but consensus was consistently achieved. The external reviewer (London based) consistently graded care more optimistically than the local reviewers which we attribute to incomplete insights to the local system. Although this might suggest a bias by the internal investigators to more stringent assessment of the system, we believe through the use of objective standards of care, and the systematic, blinded and multiparty review process we have minimized such bias.

Finally, there needs to be greater emphasis on an organization and systems approach rather than each step in isolation. Most systems focus on resource utilization efficiency (ensuring that the medical personnel throughout the system are fully utilized) rather than on “flow efficiency” (ensuring that the patient receives the most timely and effective care throughout the system) [2]. Major improvements could be achieved through simultaneous interventions at different levels of the healthcare system [38]. Policy makers and healthcare managers in this environment have already been struck by the implications and insights from this study. Further research is planned using elements of this process for routine quality assessment, and implementation of changes.

## Conclusions

Using a novel methodology and approach we have plotted the pathways followed by critically ill children from first presentation through to PICU admission or death. We highlight the complexity of that pathway and focus attention on specific issues including access to care and the importance of continuity and quality of care at each step throughout a process. Frequently occurring modifiable events were identified at each step along the pathway which can inform future interventions. Any attempts at improvement of quality of care and patient outcome should be linked to well-conducted evaluations and will need to be focussed on entire systems and not simply on components of that system.

## Supporting Information

S1 Case Studies (DOCX)

S1 Table Modifiable Factors Applied in each Facility/ EMS Assessment. (DOCX)

S2 Table Consensus Standards of Care for Paediatric Emergency Care. (DOCX)

S3 Table Comparison of Pathways to Care cohort to Western Cape population. (DOCX)

S4 Table Detailed Delay Intervals for various aspects of the Pathways to Care cases which were admitted to PICU (excludes 30 deaths prior PICU). (DOCX)

S5 Table Major impact modifiable factors and clinical review outcomes. (DOCX)

S6 Table Modifiable factors identified for primary health care facilities (GP, clinic), community health centres, and overall facilities. (DOCX)

S7 Table Modifiable factors identified for hospital level facilities. (DOCX)

S8 Table Modifiable factors identified for each type of EMS transfer. (DOCX)

S9 Table Compliance with Standards of Care for each facility and EMS level. (DOCX)

S10 Table List of variables collected in Database. (DOCX)
