# Supplementary material for: Pathways to Care for Critically Ill or Injured Children: A Cohort Study from First Presentation to Healthcare Services through to Admission to Intensive Care or Death
Source: PLoS One. 2016 Jan 5;11(1):e0145473. doi: 10.1371/journal.pone.0145473 (PMC4712128; doi:10.1371/journal.pone.0145473)
Supplement: S1 Case Studies — (DOCX) [file pone.0145473.s001.docx]

CASE STUDY 2

A 5 week old boy presented, born at 34 weeks already with an admission for sepsis at another hospital. Now with 3 days diarrhoea treated at home (informal dwelling, unemployed parents) with oral rehydration solution but worsening. Taken to CHC where seen with gastro-enteritis and moderate dehydration. Failed trial of oral rehydration, IV rehydration overnight and discharged 12 hours later (despite mother’s ongoing concerns). Acute pathway starts 36 hours later as infant returns to CHC (21h30), triaged life threatening (red), shocked, dehydrated, and hypothermic. Resuscitated with 3 x 20 ml/kg IV fluids. Possibly antibiotics given (undocumented). EMS called, delay – uncertain why – dispatch related? EMS arrived 00h00 to find child “gasping”. Intubated by EMS at CHC and transported to RXH. Arrived at RXH (00h30) and assessed as septic shock, resuscitation ongoing, for PICU. PICU admission (2h30) – required inotropes and 48 hours ventilation. Klebsiella septicaemia but with apparently good recovery.

Expert Consensus: Global Quality of Care: Poor; ICU admission: Not Avoidable; Severity at ICU admission: Avoidable.

Major Modifiable factors: CHC: Resus not done/ inadequate; Antibiotic Therapy; Inadequate Stabilization for transfer; EMS: Inadequate stabilization for transfer.

CASE STUDY 1

A 3 week old term baby girl presented with a few hours of dyspnoea, diarrhoea and vomiting - given traditional medicines at home with no effect. By midnight worsening, so driven by a neighbour to a community health centre (CHC). At the CHC the mother had great difficulty waking the reception clerk to open a folder, eventually triaged an hour later as urgent (orange) and seen by a doctor around 2h00. Assessed as dehydration, shock. Struggled to site IV, given single IV fluid bolus of 20 ml/kg followed by a maintenance infusion, no further monitoring or management documented. EMS called, arrived 70 minutes later (3h40) to find child shocked, no oxygen or IV access, no handover from CHC staff. EMS attempts to resite IV en-route failed. Arrived at Specialist Centre (RXH) at 4h37, first assessed 5h15. Referral letter left at CHC, so no record of prior management. Assessed with septic shock, hypothermia (32.8 C). Resusscitated with further fluid bolus’s, antibiotics (first received 5h30), oxygen and inotropes. Paediatric Intensive Care (PICU) bed requested – none available – monitored in EC until PICU admission at 9h00. PICU initial assessment as still shocked. Intubated and ventilated; maximal therapy for septic shock with multi-organ failure; died 48 hours later.

Expert Consensus: Global Quality of Care: Poor; ICU admission: Not Avoidable; Severity at ICU admission: Avoidable.

Major Modifiable factors: CHC: Resus not done/ inadequate; Antibiotic Therapy; Inadequate Stabilization for transfer; EMS: Inadequate stabilization for transfer.
