# Supplementary material for: Pathways to Care for Critically Ill or Injured Children: A Cohort Study from First Presentation to Healthcare Services through to Admission to Intensive Care or Death
Source: PLoS One. 2016 Jan 5;11(1):e0145473. doi: 10.1371/journal.pone.0145473 (PMC4712128; doi:10.1371/journal.pone.0145473)
Supplement: S1 Table — (DOCX) [file pone.0145473.s002.docx]

S**1 Table. Modifiable Factors Applied in each Facility/ EMS Assessment**

|  | |
| --- | --- |
| **FACILITY** | **COMMUNICATION** |
| Accessibility of Emergency Care area/ personnel | Explanation to caregiver |
| **TRIAGE** | Communication death issues |
| Inadequate assessment at triage | Other |
| Triage mechanism misses critical patient | **EMS** |
| Other | Communication with call centre at initiation of transfer |
| **INITIAL ASSESSMENT** | Communication from control to dispatched crew |
| Missing key findings (history/ clinical) | Prioritization of call out |
| Inadequate assessment/ interpretation of severity | Dispatch time delay |
| Investigations inadequate | Transfer time excessive |
| Investigations excessive | Response time delay |
| Missed/ incorrect diagnosis | Inappropriate vehicle/ crew/ equipment |
| Other | Inadequate stabilization for transfer |
| **MANAGEMENT** | Inadequate assessment before transfer |
| Delay in critical management decisions | Inadequate monitoring en route |
| Resuscitation not done/ inadequate for shock | EMS clinical management decision |
| Airway management | EMS disposal decision |
| Ventilatory management | Other |
| Circulatory management | **OPERATING THEATRE (those not covered above)** |
| Haemo-glucose test assessment & management | Anaesthetic Pre-op Assessment Inadequate |
| Antibiotic therapy | Anaesthetic Senior not called pre-op |
| Analgesia | Surgical Pre-op Assessment Inadequate |
| Temperature management | Surgical Senior not called pre-op |
| Electrolyte abnormality management | Delay pre-op |
| Trauma Immobilization | Anaesthetic technique |
| Delay in disposal decisions | Fluid Management |
| Other | Surgical technique |
| **CONSULTATION** | Delay on table |
| Inadequate supervision of junior staff | Delay in calling senior for assistance |
| No consultation to on site seniors | Recovery Process issues |
| No consultation to offsite specialists | Delay in transfer out |
| Senior review of patients (e.g. ward round) inadequate | Other |
| Delayed consultation | **DOCUMENTATION** |
| Other | Missing date/ times |
| **REFERRAL** | Missing / poorly documented information |
| Inappropriate referral destination | Other document issues |
| Communications with receiving facility | **RADIOLOGY** |
| Call/ information given to EMS about transfer | Delay awaiting radiology |
| Inappropriate referral mechanism (e.g. taxi/ private transport) | Delay in performing radiology |
| Inadequate stabilization for transfer | Delay reporting radiology |
| Ongoing monitoring/ management while awaiting transfer | Radiology findings missed/ misinterpreted |
| Referral Delay | Other |
| Other | **ADVICE TO PARENTS** |
|  | No documentation of advice given |
|  | No documentation but parents recall advice |

*PICU paediatric intensive care unit; EMS emergency medical services*

***Definitions of Impact of Modifiable Factors*** *(for each facility, transfer or ward within a hospital)*

***Major Impact*** *– factor which had clear negative impact on the outcome for the patient (worsened mortality or morbidity); directly and overwhelmingly important factor in the severity of illness/ death*

***Moderate Impact*** *– factor which on its own had minimal negative impact on the outcome but may have caused some morbidity and/ or extended the hospital/ PICU stay*

***Near Miss*** *– unplanned event that did not have major impact– but had the potential to do so - only a fortunate break in the chain of events prevented an injury, fatality or damage*

***No Defined Impact*** *– factor which has no individual or cumulative negative impact on the outcome of this or future cases*

***Not known*** *– cannot be established or estimated given facts known about scenario*
