# Supplementary material for: Pathways to Care for Critically Ill or Injured Children: A Cohort Study from First Presentation to Healthcare Services through to Admission to Intensive Care or Death
Source: PLoS One. 2016 Jan 5;11(1):e0145473. doi: 10.1371/journal.pone.0145473 (PMC4712128; doi:10.1371/journal.pone.0145473)
Supplement: S2 Table — (DOCX) [file pone.0145473.s003.docx]

**S2 Table. Consensus Standards of Care for Paediatric Emergency Care**

| **FACILITY STANDARDS** | | | |
| --- | --- | --- | --- |
|  | **CRITICAL^1^** | **IMPORTANT^1^** | **NECESSARY^1^** |
| **1. GENERAL/ TRIAGE** | 1.1. Entry to facility - eyeball assessment (5 min) | 1.2. Fast track for paeds |  |
|  | 1.3. Triage system in use | 1.7. Triage YELLOW -Mx (60 min) |  |
|  | 1.4. Triage RED - Mx by senior HCP (Immed) | 1.8. Retriage if Unseen in (2 hrs) |  |
|  | 1.5. Oxygen therapy Red in (5 min) | 1.9. Analgesia (20 min) |  |
|  | 1.6. Triage ORANGE - Mx (10 min) |  |  |
| **2. DOCUMENTATION** |  | 2.5. Doc weight | 2.1. Doc time arrival |
|  |  | 2.3. Doc time seen by HCP | 2.2. Doc time triage/ first assessed |
|  |  | 2.4. Doc time Rx commenced | 2.6. Doc time date each assess/treat |
|  |  |  | 2.7. Doc HCW name |
| **3. GASTRO** | 3.1. Gastro HGT at intial assessm (IMMED) | 3.5. Oral Rehydr Area in facil |  |
|  | 3.2. Oxygen to Shocked Gastro (IMMED) | 3.6. <=5% given ORS & reassess |  |
|  | 3.3. Doc of signs & severity | 3.7. Fails ORS @ 2 hrs then NGT |  |
|  | 3.4. ID & Doc signs shock | 3.8. Well-nourished 20 ml/kg of 1/2 DD IV |  |
|  | 3.10.Oxygen admin if >=10% | 3.9. Malnourished NGT 10 ml/kg/hr |  |
|  | 3.11.HGT check & Mx if >=10% | 3.13.Shock:Unable site IV/IO then NG 30ml/kg/hr |  |
|  | 3.12.IV or IO access |  |  |
|  | 3.14.Shock Mx 20ml/kg NS repeated |  |  |
|  | 3.15.Shock:HGT check & Mx |  |  |
|  | 3.16.Shock:2nd IV bolus & look cause |  |  |
|  | 3.17.Shock: 3rd IV bolus & call help |  |  |
|  | 3.18.Oxygen to all shocked |  |  |
| **4. RESPIRATORY** | 4.1. Airway Management (IMMED) | 4.6. Airway: Performed by competent HCP ASAP | 4.15.Croup:Steroid prescription |
|  | 4.2. ID & doc of problem airway | 4.8. ETT correct size & depth | 4.18. Pneum: Classification mod/sev/v sev |
|  | 4.7. BVM as alternative | 4.9. ETT confirmation placement | 4.25.Asthma: Dose of steroid/ b dilator |
|  | 4.3. ID & doc of severity respir distress | 4.10.Ventilator setup correct | 4.26.Asthma: Discharge information/ checks |
|  | 4.4. Basic Airway manoeuvres | 4.11.Post intubation care (5 min) |  |
|  | 4.5. Oxygen to all sats <92 or other signs | 4.12.Documentation Adv Airwy Mx |  |
|  | 4.14.Croup:Adrenaline nebs | 4.13.Croup:Doc signs severity |  |
|  | 4.16.Severe Pneumonia Assesm-Abics (30 min) | 4.17.Pneum: Doc signs pneumonia severity |  |
|  | 4.19.Pneumonia: IV appropr antibiotics in 30 min | 4.20.Pneum: Dose antibiotics correct |  |
|  | 4.21.Asthma triage- nebs (5 min) | 4.22.Asthma Doc signs severity |  |
|  |  | 4.23.Asthma: Nebulize b/dilator(5 min) |  |
|  |  | 4.24.Asthma: Steroid administration |  |
| **5. FEVER/SEPTIC SHOCK** | 5.3. Fever -Abics within (60 min) | 5.8. Doc evidence meningitis | 5.1. Fever temp at triage (IMMED) |
|  | 5.4. Septic Shock triage-IV bolus (10 min) | 5.14.Inotropes/ vasopressors if unresponsive | 5.2. Fever T >38,5-antipyretic (30 min) |
|  | 5.5. Septic Shock triage-Abics (30 min) | 5.7. Urine testing (if <3/12 or no obv cause) | 5.6. Temperature documented at triage |
|  | 5.9. Early recogn septic shock |  |  |
|  | 5.10. ID & Doc SHOCK | 5.15.Referral of s/shock to appropr facility(PICU) |  |
|  | 5.11.I V IO access early |  |  |
|  | 5.12. HGT Mx (check and treat as approp) |  |  |
|  | 5.13. Approp Abic given IV/IM |  |  |
| **6. COMA/CONVULSIONS** | 6.1. Coma/ Convuls - HGT Mx (IMMED) | 6.2. ID & Doc Level of Consciousness (GCS/AVPU) | 6.7. Neonatal Convuls phenobarbitone |
|  | 6.3. Airway Manouevres as appropr | 6.6.Convuls: Anticonvuls dose correct |  |
|  | 6.4. Oxygen administered | 6.11.Coma: Neuro-observations |  |
|  | 6.5. Convul: Anticonvulsant given appropr route | 6.9. Convul: Post convuls - Mx |  |
|  | 6.12. Coma: Brain imaging referral | 6.10.Coma:Anticonvulsant as appropr |  |
|  | 6.13.Coma: Referral facility as appropr |  |  |
| **7. TRAUMA** | 7.2. IV/ IO insertion if signs shock (IMMED) | 7.1. Trauma Assess- Analgesia (ASAP) | 7.5. Remain with parent/ carer |
| **POLYTRAUMA (PT)** | 7.3. Bolus IV/IO fluid to shocked pt (IMMED) | 7.4. PT Analgesia Mx | 7.9. PT: NPO |
| **Head Injury (HI)** | 7.7. PT: Bolus 10 ml/kg IV R/L or N/S ASAP | 7.6. PT Spinal Immobilization |  |
| **Orthopaedics (Ortho)** | 7.14. HI: CT Brain requir’d ID and expedite | 7.8. PT Blood transfusion after 40ml/kg (30 min) |  |
| **BURNS** | 7.15.HI Airway Manouevres as appropr | 7.12.HI Head Injury document GCS IMMED (ASAP) | 7.23.Ortho :Procedural Sedation all interventions |
|  | 7.16.HI Intubation where indic & appropr | 7.13.HI ID & Doc Level of Consciousness (20 min) | 7.26.Documentation of burn wound : |
|  | 7.19.HI Transfer approp as per HI Guidelines | 7.17.HI BVM as an alternative | 7.26.1. region & area of burn % |
|  | 7.20.HI Neurosurgical review ASAP | 7.18.HI Neuro-observation | 7.26.2. Depth of burn |
|  | 7.28.BURN: IV fluids as per Parklands 3.5xWTx% | 7.21.Ortho: Immobilz & redn fractures ASAP | 7.26.3. Time of burn |
|  | 7.29.BURN: Search inhalation burns | 7.22.Ortho: Open #/Complic Wound – Abics (30 min) | 7.26.4. Mechanism of burn |
|  | 7.30.BURN: Inhal Burns – early intubation | 7.24.Severe Burns - Occlussive Dressing (ASAP) |  |
|  |  | 7.25.Burns: Analgesia severe burns (20 min) |  |
|  |  | 7.27.Burns: Mx according to SABSG (2 hrs) |  |
|  |  | 7.31.Burns: Escharotomy where appropr |  |
|  |  | 7.32.Burns: Referral as per SABSG or RXCH criter |  |
|  |  | 7.10.PT: NAI consideration |  |
|  |  | 7.11.PT: ATT open wounds |  |
| **8. Referral** |  | 8.1. Early EMS contact by PHC/ CHC level | 8.3. Referral letter contents |
|  |  | 8.2. Referral communications for critical paeds |  |
|  |  | 8.4. Acceptance of any critical child by L2/3 |  |
|  |  | 8.6. EMS communication to rec facility |  |
|  |  | 8.5. Facility handover to/from EMS |  |
| **9. ICU Referral** | 9.1. ICU request to ICU assessment | 9.3. ICU request (non-ventil) to ICU bed ( 4 hrs) | 9.6. Monitoring Ventil/ Crit Pt: nurse ratio 1:1 |
|  | 9.2. ICU request (ventil) to ICU bed | 9.5. Specialist consult/review when nec (15 min) | 9.7. Monitoring of vitals every 15 m |
|  | 9.4. Maximum ventilation outside PICU (24rs) | 9.8. Monitor/ventil equipm outside ICU |  |
|  |  |  |  |
| **EMS STANDARDS** | | | |
|  | **CRITICAL^1^** | **IMPORTANT^1^** | **NECESSARY^1^** |
| **1. GENERAL** | 1.1. P1 Calls EMS call-scene (15 min) |  | 1.6. Referral communications for critical paeds |
|  | 1.2. Arrival to Oxygen for RED (IMMED) | 1.10.EMS route child to closest approp facil | 1.9. EMS communication to rec facility |
|  | 1.3. Triage system in use |  | 1.12.EMS handover rec/ del facility |
|  | 1.4. EMS SATS RED treated by ALS |  |  |
|  | 1.5. EMS Paeds approp resus equipment |  |  |
|  | 1.7. EMS PFS for interfacility red transfer |  |  |
|  | 1.8. EMS ALS if no PFS |  |  |
|  | 1.11.Acceptance of any critical child L2/3 |  |  |
| **2. EMS DOCUMENTATION** |  | 2.1. Doc time call received |  |
|  |  | 2.2. Doc time EMS team dispatched |  |
|  |  | 2.3. Doc time arrival |  |
|  |  | 2.4. Doc EMS time depart scene/ref facility |  |
|  |  | 2.5. Doc EMS time destin facil |  |
|  |  | 2.6. Doc EMS management |  |
|  |  | 2.7. Doc EMS HCW name |  |
| **3. EMS GASTRO** | 3.1. Gastro HGT at intial assessm (IMMED) | 3.3. Doc of signs & severity |  |
|  | 3.2. Oxygen to Shocked Gastro (IMMED) | 3.5. Well nourished 20 ml/kg 1/2 DD IV |  |
|  | 3.4. ID & Doc signs shock | 3.11.2nd IV bolus & look cause |  |
|  | 3.6. Oxygen admin if >=10% | 3.12.3rd IV bolus & call help |  |
|  | 3.7. HGT check & Mx if >=10% |  |  |
|  | 3.8.Shock: IV or IO access |  |  |
|  | 3.9. Shock Mx 20ml/kg NS repeated |  |  |
|  | 3.10.SHOCK:HGT check & Mx |  |  |
| **4. EMS RESPIRATORY** | 4.2. ID & Doc of Respiratory Distress | 4.1. Asthma asses- nebs (5min) | 4.7. ETT correct size & depth |
| **Advanced Airway Management** | 4.3. Basic Airway Manoeuvres | 4.8. ETT confirmation placement | 4.9. Ventilator setup correct |
|  | 4.4. Oxygen to all appropr |  | 4.10.Post intubation care |
|  | 4.5. Airwy: Performed by competent HCP ASAP |  |  |
|  | 4.6. BVM as alternative |  |  |
| **5. EMS FEVER/ SEPTIC SHOCK** | 5.1. Septic Shock triage-IV bolus (10min) |  | 4.11.Documentation Adv Airwy Mx |
|  | 5.2. Early recogn septic shock |  |  |
|  | 5.3. ID & Doc SHOCK |  |  |
|  | 5.4. IV IO access early |  |  |
|  | 5.5. HGT Mx (check and treat as approp) |  |  |
| **6. EMS COMA/CONVULS** | 6.1. Coma/ Convuls HGT Mx (IMMED) | 6.2. ID & Doc Level of Consciousness(GCS/AVPU) | 6.7. Post convuls Mx |
|  | 6.3. Airway Manouevres as appropr | 6.5. Conv: Anticonvulsant given appropr route |  |
|  | 6.4. Oxygen administered | 6.6. Conv: Anticonvuls dose correct |  |
|  |  | 6.8. Conv: ID & Doc Level of Consciousness |  |
|  |  | 6.9. Conv: Airway Manouevres as appropr |  |
|  |  | 6.10.Coma: Anticonvulsant as appropr |  |
|  |  | 6.11.Coma: Neuro-observations |  |
| 7. EMS TRAUMA | 7.2. IV/ IO insertion if signs shock (IMMED) | 7.1. Trauma Assess- Analgesia (10 min) | 7.6. PT: Remain with parent/ carer |
| POLYTRAUMA (PT) | 7.3. Bolus IV/IO fluid to shocked pt (IMMED) | 7.4. Head Injury documentn GCS (IMMED) | 7.18.Documentation of burn wound : |
| Head Injury (HI) | 7.8. PT: Bolus 10 ml/kg IV R/L or N/S ASAP | 7.5. Severe Burns - Occlussive Dressing (10 min) | 7.18.1. region & area of burn % |
| Orthopaedics (Ortho) | 7.11.HI: Airway Manouevres as appropr | 7.7. PT: Spinal Immobilization | 7.18.2. Depth of burn |
|  | 7.13.HI: BVM as an alternative | 7.9. HI: Head Injury documentn GCS (IMMED) | 7.18.3. Time of burn |
|  | 7.12.HI: Intubation where indic & appropr | 7.10.HI: ID & Doc Level of Consciousness | 7.18.4. Mechanism of burn |
|  | 7.14 HI: .Transfer approp as per HI Guidelines | 7.15.Ortho: Immobilz & redn fractures ASAP |  |
|  |  | 7.16.Severe Burns – Occl. Dressing (ASAP) |  |
|  |  | 7.17.Analgesia severe burns (20 min) |  |
|  |  |  |  |

^1^ *Grading of standards: critical standards (life threatening essential issues of absolute and time critical importance); important standards (issues that need to be addressed but perhaps without the acuity and clear link to outcome of individual cases); necessary standards (issues such as documentation and classification that are necessary in the system but have less impact on individual outcome)*

*# fracture;   Abics antibiotics;   ALS advanced life support;   ATT anti tetanus toxoid;   AVPU awake/ verbal response/ pain response/ unresponsive scale;   BVM bag valve mask;   CHC community health centre;   Doc document;   EMS emergency medical services;   ETT endo-tracheal tube;   Gastro gastroenteritis ;   GCS Glasgow come scale;   HCP health care provider;   HGT haemo-glucose test;   HI head injury;   ICU intensive care unit;   ID identification;   IO intra-osseous;   IV intra venous;  Mx management;  NAI non accidental injury;   Neb nebulizer;   NG naso-gastric;   NPO nil per os;   NS normal saline;   ORS oral rehydration solution;   PFS paediatric flying squad;   PHC primary health care;   RL ringers lactate;   Rx treatment;   SABSG South African Burns Society Guidelines ;   Sats saturation (pulse oximetry)*
