# Supplementary material for: Pathways to Care for Critically Ill or Injured Children: A Cohort Study from First Presentation to Healthcare Services through to Admission to Intensive Care or Death
Source: PLoS One. 2016 Jan 5;11(1):e0145473. doi: 10.1371/journal.pone.0145473 (PMC4712128; doi:10.1371/journal.pone.0145473)
Supplement: S3 Table — (DOCX) [file pone.0145473.s004.docx]

**S3 Table. Comparison of Pathways to Care cohort to Western Cape population**

| **Indicator** | **Pathways to Care Cases** | **Western Cape^a^** |
| --- | --- | --- |
| % living in informal dwellings | 44.7 | 22.0 |
| Household Annual Income^b^ | 88.3% < $7400  68.9% < $3700 | Mean $17 654 |
| Child Support Grant in place^c^ | 53% | 38.1% |
| Single parent | 58% | - |
| **Mother** | | |
| Mother’s age | Mean 28.9 (SD 6.9) | - |
| Maternal Unemployment rate | 65.6% | 21.4% |
| Mother’s schooling Grade 11-12 or higher | 53% | 43% |
| **Father** | | |
| Father’s age | Mean 33.0 (SD 8.5) | - |
| Father’s unemployment rate | 24.5% | 21.4% |
| Father’s highest schooling | 49% Grade 11-12 or higher | 43% |
| Both parents unemployed | 50/282 (17.7%) | 11% |
| **Amenities** | | |
| Cooking – electric | 89.4% | 86.9% |
| Heating – electric | 65.2% | 63.5% |
| Lighting – electric | 91.5% | 93.4% |
| Water – on site | 74.8% | 88.4% |
| Sanitation – toilet on site | 86.1% | 92.0% |
| **Assets** | | |
| Radio | 63.5% | 69.2% |
| Television | 88.3% | 85.5% |
| Satellite Dish | 17.4% | 30.6% |
| Fridge | 74.1% | 80.5% |
| Stove | 93.6% | 90.2% |
| Washing Machine | 37.2% | 57.6% |
| Motor Vehicle | 17% | 43.6% |
| Cellular Phone | 91.5% | 88.9% |
| Computer | 12.4% | 34.4% |
| **Distance & transport** | | |
| Distance to nearest health facility | 3.5 km (SD 6.4) | 14% > 30 minutes away |
| Distance to nearest 24hr facility | 10.5 km (SD 17.5) | - |
| Means of transport | 71% walk, 22% taxi | - |

^a^ *Census 2011 Municipal Report Western Cape [20]*

^b^ *exchange rate based on mean $:ZAR for study period of 8.1*

*^c^ South Africa has a system nationally of a monthly child support grant to eligible parents of $35 per child.*
