# Supplementary material for: Pathways to Care for Critically Ill or Injured Children: A Cohort Study from First Presentation to Healthcare Services through to Admission to Intensive Care or Death
Source: PLoS One. 2016 Jan 5;11(1):e0145473. doi: 10.1371/journal.pone.0145473 (PMC4712128; doi:10.1371/journal.pone.0145473)
Supplement: S4 Table — (DOCX) [file pone.0145473.s005.docx]

**S4 Table. Detailed Delay Intervals for various aspects of the Pathways to Care cases which were admitted to PICU (excludes 30 deaths prior PICU)**

| **Time Delays** | | | **Medical** | | **Trauma** | | **Overall** | |
| --- | --- | --- | --- | --- | --- | --- | --- | --- |
|  |  |  | **N** | **Median (IQR)** | **N** | **Median (IQR)** | **N** | **Median (IQR)** |
| Onset illness to first presentation (days) | | | 218 | 2.0 (0.0-3.0) | 34 | 0.0 (0.0-0.0) | 252 | 1.0 (0.0-3.0) |
| First presentation to RCWMCH (hours) | Within Cape Town (n=227) |  | 201 | 4.1 (1.8-7.4) | 26 | 1.5 (0.8-2.3) | 227 | 3.6 (1.5-6.8) |
|  | Number of facilities visited prior to RCWMCH for Cape Town cases | 0 | 37 | 0.0 (0.0-0.0) | 12 | 0.8 (0.4-1.2) | 49 | 0.0 (0.0-0.7) |
|  |  | 1 | 123 | 4.2 (2.5-5.9) | 13 | 2.3 (1.8-3.2) | 136 | 3.9 (2.3-5.8) |
|  |  | 2 | 35 | 10.2 (5.7-52.4) | 1 | 19.3 (19.3-19.3) | 36 | 11.3 (5.9-52.3) |
|  |  | >=3 | 6 | 36.8 (18.1-95.0) | 0 | - | 6 | 36.8 (18.1-95.0) |
|  | Outside of Cape Town (n=25) | | 17 | 34.9 (13.3-73.6) | 8 | 7.6 (4.5-10.1) | 25 | 20.4 (6.3-49.0) |
|  | Overall presentation - RCWMCH | | 218 | 4.4 (1.9-9.2) | 34 | 1.9 (1.0-5.2) | 252 | 4.2 (1.7-8.9) |
| EMS Intervals^a^ (minutes) | Dispatch | | 237 | 7.0 (2-31) | 55 | 3.0 (0-6) | 292 | 5.0 (2-30) |
|  | En Route1 | |  | 13.0 (5-20) |  | 10.0 (5-17) |  | 12.0 (5-19) |
|  | On Scene | |  | 24.0 (14-40) |  | 35.0 (15-57) |  | 25.0 (14-43) |
|  | En Route2 | |  | 19.0 (13-30) |  | 19.5 (14-43) |  | 19.0 (13-31) |
|  | EMS Total | |  | 86.0 (56-124) |  | 80.0 (48-128) |  | 86.0 (54-124) |
| RCWMCH arrival to PICU (hours) | Direct PICU | | 31 | - | 1 | - | 32 | - |
|  | ED - PICU | | 117 | 4.1 (2.8-5.8) | 18 | 3.3 (2.0-5.0) | 135 | 4.1 (2.7-5.8) |
|  | ED – Ward - PICU | | 44 | 23.4 (14.7-49.9) | 1 | 113.8 (113.8-113.8) | 45 | 24.5 (15.9-50.4) |
|  | ED - Ward – Ward –PICU^b^ | | 12 | 47.3 (28.3-77.8) | 0 | - | 12 | 47.3 (28.3-77.8) |
|  | ED – OT- PICU | | 7 | 7.3 (3.1-8.8) | 10 | 6.9 (6.5-11.3) | 17 | 7.2 (6.5-8.8) |
|  | ED - Ward – OT -PICU ^c^ | | 7 | 30.0 (19.3-54.8) | 4 | 27.5 (8.9-46.0) | 11 | 30.0 (17.3-51.3) |
|  | Overall RCWMCH to PICU | | 218 | 5.0 (2.4-15.9) | 34 | 5.5 (3.1-8.1) | 252 | 5.0 (2.5-12.9) |
| PICU bed request to PICU admission (hours) | | | 218 | 2.5 (1.5-4.0) | 34 | 3.0 (1.3-4.0) | 252 | 2.5 (1.5-4.0) |
| First presentation to PICU (hours) | Within Cape Town | | 201 | 13.3 (7.1-41.6) | 26 | 9.3 (6.3-16.0) | 227 | 11.9 (6.8-34.8) |
|  | Outside of Cape Town | | 17 | 40.5 (20.4-73.6) | 8 | 10.3 (6.4-23.9) | 25 | 30.0 (9.8-56.7) |

*IQR interquartile range; RCWMCH Red Cross War Memorial Children’s Hospital; EMS emergency medical services; PICU paediatric intensive care unit; OT operating theatre*

*^a^ EMS delays are analysed per EMS trip and there were variable numbers of trips per case thus n=292*

^b^ *All but one case involved overnight admission from RCWMCH Emergency Department to an overnight/ short stay ward and then admission to a medical ward. A single case was referred as a “step-down” to a regional hospital and then deteriorated and returned to RCWMCH*

^c^ *2 (medical) cases returned to a ward from OT and were subsequently admitted to PICU*
