# Supplementary material for: Pathways to Care for Critically Ill or Injured Children: A Cohort Study from First Presentation to Healthcare Services through to Admission to Intensive Care or Death
Source: PLoS One. 2016 Jan 5;11(1):e0145473. doi: 10.1371/journal.pone.0145473 (PMC4712128; doi:10.1371/journal.pone.0145473)
Supplement: S5 Table — (DOCX) [file pone.0145473.s006.docx]

**S5 Table. Major impact modifiable factors and clinical review outcomes (n=477)^a^**

| **Global quality of care received poor (n= 57 children)** | | | **Severity of illness avoidable/potentially avoidable (n=185 children)** | | | **PICU avoidable/potentially avoidable (n=61 children)** | | | **Death avoidable/potentially avoidable (n=17 children)** | | |
| --- | --- | --- | --- | --- | --- | --- | --- | --- | --- | --- | --- |
| **Modifiable Factors** | ***n= 233*** | **%** | **Modifiable Factors** | ***n= 362*** | **%** | **Modifiable Factors** | ***n= 170*** | **%** | **Modifiable Factors** | ***n= 105*** | **%** |
| Inadequate assessment/ interpretation of severity | 39 | 16.7 | Inadequate assessment/ interpretation of severity | 48 | 13.3 | Inadequate assessment/ interpretation of severity | 28 | 16.5 | Inadequate assessment/ interpretation of severity | 17 | 16.2 |
| Resuscitation not done/ inadequate for shocked patient | 30 | 12.9 | Resuscitation not done/ inadequate for shocked patient | 46 | 12.7 | Resuscitation not done/ inadequate for shocked patient | 18 | 10.6 | Resuscitation not done/ inadequate for shocked patient | 11 | 10.5 |
| Accessibility of Emergency Care area/ personnel | 13 | 5.6 | Delay in critical management decisions | 21 | 5.8 | Delay in critical management decisions | 10 | 5.9 | Circulatory management | 8 | 7.6 |
| Circulatory management | 13 | 5.6 | Accessibility of Emergency Care area/ personnel | 21 | 5.8 | Accessibility of Emergency Care area/ personnel | 9 | 5.3 | Missing key findings (history/ clinical) | 6 | 5.7 |
| Missing key findings (history/ clinical) | 11 | 4.7 | Circulatory management | 20 | 5.5 | Missing key findings (history/ clinical) | 9 | 5.3 | EMS: Response time delay | 6 | 5.7 |
| Delay in critical management decisions | 10 | 4.3 | Inappropriate vehicle/ crew/ equipment | 17 | 4.7 | Circulatory management | 8 | 4.7 | Delay in critical management decisions | 5 | 4.8 |
| Antibiotic therapy | 9 | 3.9 | Referral Delay | 16 | 4.4 | Referral Delay | 7 | 4.1 | Referral Delay | 5 | 4.8 |

*PICU paediatric intensive care unit; EMS emergency medical services*

*^a^ total of 3212 modifiable factors were identified for the entire cohort (comprising 477 (14.95) major, 1826 (56.9%) moderate, 44 (1.4%) near miss, 290 (9.0%) no defined impact and 575 (17.9%) unknown impact modifiable factors)*
