# Supplementary material for: Pathways to Care for Critically Ill or Injured Children: A Cohort Study from First Presentation to Healthcare Services through to Admission to Intensive Care or Death
Source: PLoS One. 2016 Jan 5;11(1):e0145473. doi: 10.1371/journal.pone.0145473 (PMC4712128; doi:10.1371/journal.pone.0145473)
Supplement: S6 Table — (DOCX) [file pone.0145473.s007.docx]

**S6 Table. Modifiable factors identified for primary health care facilities (GP, clinic), community health centres, and overall facilities**

| **MAJOR Modifiable Factors (top 10)** | **N(% of top 10)** | **MODERATE Modifiable Factors (top 10)** | **N(% of top 10)** |
| --- | --- | --- | --- |
| **General Practitioner (n=22)** | | | |
| Inadequate assessment/ interpretation of severity | 8(26.7%) | Inadequate assessment/ interpretation of severity | 4(16.7%) |
| Inappropriate referral mechanism (e.g.. taxi/ private transport) | 8(26.7%) | Inappropriate referral mechanism (e.g.. taxi/ private transport) | 3(12.5%) |
| Resuscitation not done/ inadequate for shocked patient | 5(16.7%) | Communications with receiving facility | 3(12.5%) |
| Missing key findings (history/ clinical) | 2(6.7%) | Accessibility of Emergency Care area/ personnel | 2(8.3%) |
| No consultation to offsite specialists | 2(6.7%) | Missed/ incorrect diagnosis | 2(8.3%) |
| Inappropriate referral destination | 2(6.7%) | Explanation to caregiver | 2(8.3%) |
| Delay in critical management decisions | 1(3.3%) | Resuscitation not done/ inadequate for shocked patient | 2(8.3%) |
| Antibiotic therapy | 1(3.3%) | Missing key findings (history/ clinical) | 2(8.3%) |
| Communications with receiving facility | 1(3.3%) | No consultation to offsite specialists | 2(8.3%) |
| - |  | Antibiotic therapy | 2(8.3%) |
| **City Health Clinic (n=57)** | | | |
| Accessibility of Emergency Care area/ personnel | 12(23.1%) | Accessibility of Emergency Care area/ personnel | 15(18.8%) |
| Inadequate assessment at triage | 10(19.2%) | Inadequate assessment at triage | 11(13.8%) |
| Inadequate assessment/ interpretation of severity | 8(15.4%) | Antibiotic therapy | 11(13.8%) |
| Resuscitation not done/ inadequate for shocked patient | 7(13.5%) | Ventilatory Management | 9(11.3%) |
| Circulatory management | 4(7.7%) | Explanation to caregiver | 9(11.3%) |
| Missing key findings (history/ clinical) | 3(5.8%) | Inadequate assessment/ interpretation of severity | 8(10.0%) |
| Missed/ incorrect diagnosis | 3(5.8%) | Missing key findings (history/ clinical) | 5(6.3%) |
| Consultation inadequate | 2(3.8%) | Delay in disposal decisions | 4(5.0%) |
| Inappropriate referral mechanism (e.g.. taxi/ private transport) | 2(3.8%) | Communications with receiving facility | 4(5.0%) |
| Investigations inadequate | 1(1.9%) | Ongoing monitoring/ management while awaiting transfer | 4(5.0%) |
| **24 Hour Community Health Centre (CHC) (n=103)** | | | |
| Resuscitation not done/ inadequate for shocked patient | 25(25.5%) | Antibiotic therapy | 2(14.1%) |
| Inadequate assessment/ interpretation of severity | 17(17.3%) | Inadequate assessment/ interpretation of severity | 24(13.6%) |
| Circulatory management | 14(14.3%) | Explanation to caregiver | 24(13.6%) |
| Accessibility of Emergency Care area/ personnel | 10(10.2%) | Accessibility of Emergency Care area/ personnel | 22(12.4%) |
| Missing key findings (history/ clinical) | 6(6.1%) | Ongoing monitoring/ management while awaiting transfer | 19(10.7%) |
| Delay in critical management decisions | 6(6.1%) | Triage | 15(8.5%) |
| Ongoing monitoring/ management while awaiting transfer | 6(6.1%) | Missing key findings (history/ clinical) | 14(7.9%) |
| Antibiotic therapy | 5(5.1%) | Call/ information given to EMS about transfer | 12(6.8%) |
| Referral Delay | 5(5.1%) | Temperature management | 11(6.2%) |
| Triage | 4(4.1%) | Circulatory management | 11(6.2%) |
| **All Facilities (including hospitals) (n=612)** | | | |
| Inadequate assessment/ interpretation of severity | 59(21.4%) | Ongoing monitoring/ management while awaiting transfer | 171(18.5%) |
| Resuscitation not done/ inadequate for shocked patient | 59(21.4%) | Referral Delay | 153(16.6%) |
| Delay in critical management decisions | 28(10.1%) | Antibiotic therapy | 117(12.7%) |
| Accessibility of Emergency Care area/ personnel | 28(10.1%) | Delay in critical management decisions | 91(9.8%) |
| Circulatory management | 24(8.7%) | Delay in disposal decisions | 78(8.4%) |
| Referral Delay | 20(7.2%) | Inadequate assessment/ interpretation of severity | 74(8.0%) |
| Antibiotic Therapy | 17(6.2%) | Explanation to caregiver | 72(7.8%) |
| Missing key findings (history/ clinical) | 15(5.4%) | Accessibility of Emergency Care area/ personnel | 71(7.7%) |
| Inadequate assessment at triage | 15(5.4%) | Other - specify | 53(5.7%) |
| Missed/ incorrect diagnosis | 11(4.0%) | Inadequate assessment at triage | 44(4.8%) |

*EMS Emergency Medical Services*

*Modifiable Factor Impact: Major – factor which had clear negative impact on the outcome for the patient (worsened mortality or morbidity); directly and overwhelmingly important factor in the severity of illness/ death; Moderate – factor which on its own had minimal negative impact on the outcome but may have caused some morbidity and/ or extended the hospital/ PICU stay*
