# Supplementary material for: Pathways to Care for Critically Ill or Injured Children: A Cohort Study from First Presentation to Healthcare Services through to Admission to Intensive Care or Death
Source: PLoS One. 2016 Jan 5;11(1):e0145473. doi: 10.1371/journal.pone.0145473 (PMC4712128; doi:10.1371/journal.pone.0145473)
Supplement: S7 Table — (DOCX) [file pone.0145473.s008.docx]

**S7 Table. Modifiable factors identified for hospital level facilities**

| **MAJOR Modifiable Factors (top 10)** | **N(% of top 10)** | **MODERATE Modifiable Factors (top 10)** | **N(% of top 10)** |
| --- | --- | --- | --- |
| **District Hospital (n=45)** | | | |
| Resuscitation not done/ inadequate for shocked patient | 7(27.0%) | Ongoing monitoring/ management while awaiting transfer | 13(16.7%) |
| Inadequate assessment/ interpretation of severity | 6(23.1% | Delay in disposal decisions | 12(15.4%) |
| Delay in critical management decisions | 3(11.5%) | Antibiotic therapy | 9(11.5%) |
| Ventilatory management | 2(7.7%) | Explanation to caregiver | 8(10.3%) |
| Antibiotic therapy | 2(7.7%) | Inadequate assessment/ interpretation of severity | 7(8.9%) |
| Referral Delay | 2(7.7%) | Accessibility of Emergency Care area/ personnel | 7(8.9%) |
| Accessibility of Emergency Care area/ personnel | 1(2.2%) | Referral Delay | 6(7.7%) |
| Inadequate assessment at triage | 1(2.2%) | Circulatory management | 6(7.7%) |
| Missing key findings (history/ clinical) | 1(2.2%) | Analgesia | 5(6.4%) |
| Investigations inadequate | 1(2.2%) | Ventilatory Management | 5(6.4%) |
| **Regional Hospital (n=50)** | | | |
| Accessibility of Emergency Care area/ personnel | 4(23.5%) | Delay in disposal decisions | 8(15.1%) |
| Triage | 2(11.8%) | Delay in critical management decisions | 8(15.1%) |
| Inadequate assessment/ interpretation of severity | 2(11.8%) | Ongoing monitoring/ management while awaiting transfer | 7(13.2%) |
| Missed/ incorrect diagnosis | 2(11.8%) | Accessibility of Emergency Care area/ personnel | 5(9.4%) |
| Delay in critical management decisions | 2(11.8%) | Other - specify | 5(9.4%) |
| Antibiotic therapy | 2(11.8%) | Antibiotic therapy | 5(9.4%) |
| Inadequate assessment at triage | 1(5.9%) | Call/ information given to EMS about transfer | 4(7.6%) |
| Airway Management | 1(5.9%) | Inadequate assessment at triage | 4(7.6%) |
| Referral Delay | 1(5.9%) | Airway Management | 4(7.6%) |
| - |  | Ventilatory Management | 3(5.7%) |
| **RCWMCH Emergency Department (n=241)** | | | |
| Resuscitation not done/ inadequate for shocked patient | 11(18.9%) | Ongoing monitoring/ management while awaiting transfer | 106(23.9%) |
| Referral Delay | 11(18.9%) | Referral Delay | 97(21.8%) |
| Inadequate assessment/ interpretation of severity | 9(15.5%) | Antibiotic therapy | 51(11.5%) |
| Delay in critical management decisions | 7(12.0%) | Delay in critical management decisions | 47(10.6%) |
| Antibiotic therapy | 4(6.8%) | Delay in disposal decisions | 34(7.6%) |
| Delayed consultation | 4(6.8%) | Triage | 27(6.0%) |
| Inappropriate referral destination | 4(6.8%) | Explanation to caregiver | 23(5.1%) |
| Ongoing monitoring/ management while awaiting transfer | 4(6.8%) | Delayed consultation | 23(5.1%) |
| Missing key findings (history/ clinical) | 2(3.4%) | Inadequate assessment/ interpretation of severity | 19(4.2%) |
| Airway Management | 2(3.4%) | Accessibility of Emergency Care area/ personnel | 16(3.6%) |
| **RCWMCH Ward (n=74)** | | | |
| Inadequate assessment/ interpretation of severity | 8(26.7%) | Referral Delay | 35(28.0%) |
| Delay in critical management decisions | 8(26.7%) | Delay in critical management decisions | 25(20.0%) |
| Resuscitation not done/ inadequate for shocked patient | 3(10.0%) | Ongoing monitoring/ management while awaiting transfer | 22(17.6%) |
| Circulatory management | 2(6.7%) | Delayed consultation | 8(3.2%) |
| Blood Sugar assessment & management | 2(6.7%) | Delay in disposal decisions | 8(3.2%) |
| Antibiotic therapy | 2(6.7%) | Ventilatory Management | 6(4.8%) |
| Delay in disposal decisions | 2(6.7%) | Inadequate assessment/ interpretation of severity | 6(4.8%) |
| Investigations inadequate | 1(3.3%) | Antibiotic therapy | 6(4.8%) |
| Temperature Management | 1(3.3%) | Senior review of patients (e.g.. ward round) inadequate | 5(4.0%) |
| Electrolyte abnormality Management | 1(3.3%) | Circulatory management | 4(2.4%) |
| **RCWMCH Operating Theatre (n=29)** | | | |
| Anaesthetic technique | 1(100.0%) | Delay pre-op | 6(50.0%) |
| - |  | Anaesthetic Pre-op Assessment Inadequate | 2(16.7%) |
|  |  | Anaesthetic technique | 1(8.3%) |
|  |  | Airway Management | 1(8.3%) |
|  |  | Surgical Pre-op Assessment Inadequate | 1(8.3%) |
|  |  | Surgical technique | 1(8.3%) |

*RCWMCH Red Cross War Memorial Children’s Hospital; EMS Emergency Medical Services*

*Modifiable Factor Impact: Major – factor which had clear negative impact on the outcome for the patient (worsened mortality or morbidity); directly and overwhelmingly important factor in the severity of illness/ death; Moderate – factor which on its own had minimal negative impact on the outcome but may have caused some morbidity and/ or extended the hospital/ PICU stay*
