# Supplementary material for: Pathways to Care for Critically Ill or Injured Children: A Cohort Study from First Presentation to Healthcare Services through to Admission to Intensive Care or Death
Source: PLoS One. 2016 Jan 5;11(1):e0145473. doi: 10.1371/journal.pone.0145473 (PMC4712128; doi:10.1371/journal.pone.0145473)
Supplement: S8 Table — (DOCX) [file pone.0145473.s009.docx]

**S8 Table. Modifiable factors identified for each type of EMS transfer**

| **MAJOR Modifiable Factors (top 10)** | | **N(% of top 10)** | | **MODERATE Modifiable Factors (top 10)** | | **N(% of top 10)** |
| --- | --- | --- | --- | --- | --- | --- |
| **Primary (Home/ Scene) (n= 57 transfers)** | | | | | | |
| Inappropriate vehicle/ crew/ equipment | 4(30.8%) | | Inappropriate vehicle/ crew/ equipment | | 26(31.3%) | |
| Circulatory management | 2(15.4%) | | Inadequate assessment before transfer | | 13(15.7%) | |
| Response time delay | 2(15.4%) | | Inadequate stabilization for transfer | | 9(10.8%) | |
| EMS disposal decision | 1(7.7%) | | Inadequate assessment/ interpretation of severity | | 7(8.4%) | |
| Inadequate assessment/ interpretation of severity | 1(7.7%) | | Explanation to caregiver | | 6(7.2%) | |
| Inadequate assessment at triage | 1(7.7%) | | EMS disposal decision | | 5(6.0%) | |
| EMS: Inadequate assessment before transfer | 1(7.7%) | | Inadequate monitoring en route | | 5(6.0%) | |
| Other | 1(7.7%) | | Analgesia | | 4(4.8%) | |
|  |  | | Dispatch time delay | | 4(4.8%) | |
|  |  | | Ventilatory Management | | 4(4.8%) | |
| **Interfacility Transfer (non PFS) (n= 143 transfers)** | | | | | | |
| Inappropriate vehicle/ crew/ equipment | 12(25.0%)) | | Explanation to caregiver | | 52(22.8%) | |
| Response time delay | 12(25.0%)) | | Inappropriate vehicle/ crew/ equipment | | 37(16.2%) | |
| Inadequate stabilization for transfer | 7(14.6%) | | Inadequate monitoring en route | | 35(15.4%) | |
| Dispatch time delay | 5(10.4%) | | Inadequate assessment before transfer | | 27(11.8%) | |
| Inadequate assessment before transfer | 3(6.3%) | | Response time delay | | 26(11.4%) | |
| Communication with call centre at initiation of transfer | 3(6.3%) | | Temperature management | | 13(5.7%) | |
| Circulatory management | 2(4.2%) | | Communication with call centre at initiation of transfer | | 12(5.3%) | |
| Inadequate assessment/ interpretation of severity | 2(4.2%) | | Ventilatory Management | | 11(4.8%) | |
| Transfer time excessive | 1(2.1%) | | Transfer time excessive | | 8(3.5%) | |
| Inadequate monitoring en route | 1(2.1%) | | Inadequate stabilization for transfer | | 7(3.1%) | |
| **Paediatric Flying Squad (n= 36 transfers)** | | | | | | |
| Response time delay | 5(25.0%) | | Response time delay | | 15(37.5%) | |
| Blood-sugar assessment & management | 3(15.0%) | | Transfer time excessive | | 6(15.0%) | |
| Inadequate stabilization for transfer | 3(15.0%) | | Explanation to caregiver | | 5(12.5%) | |
| Inadequate assessment before transfer | 2(10.0%) | | Temperature management | | 3(7.5%) | |
| Inappropriate vehicle/ crew/ equipment | 2(10.0%) | | Inadequate monitoring en route | | 3(7.5%) | |
| Airway Management | 1(5.0%) | | Inadequate assessment before transfer | | 2(5.0%) | |
| Ventilatory Management | 1(5.0%) | | Communications with receiving facility | | 2(5.0%) | |
| Circulatory management | 1(5.0%) | | Blood-sugar assessment & management | | 2(5.0%) | |
| Communications with receiving facility | 1(5.0%) | | Inappropriate vehicle/ crew/ equipment | | 1(2.5%) | |
| Dispatch time delay | 1(5.0%) | | Ventilatory Management | | 1(2.5%) | |
| O**verall Emergency Medical Services (n=292 transfers)** | | | | | | |
| Inappropriate vehicle/ crew/ equipment | 20(25.6%) | | Explanation to caregiver | | 67(19.6%) | |
| Response time delay | 19(24.4%) | | Inappropriate vehicle/ crew/ equipment | | 65(19.0%) | |
| Inadequate stabilization for transfer | 10(12.8%) | | Inadequate monitoring en route | | 44(12.9%) | |
| Inadequate assessment before transfer | 6(7.7%) | | Response time delay | | 43(12.6%) | |
| Dispatch time delay | 6(7.7%) | | Inadequate assessment before transfer | | 43(12.6%) | |
| Circulatory management | 5(6.4%) | | Temperature management | | 18(5.3%) | |
| Communication with call centre at initiation of transfer | 4(5.1%) | | Ventilatory Management | | 17(5.0%) | |
| Blood-sugar assessment & management | 3(3.9%) | | Inadequate stabilization for transfer | | 17(5.0%) | |
| Inadequate assessment/ interpretation of severity | 3(3.9%) | | Inadequate assessment/ interpretation of severity | | 14(4.1%) | |
| Inadequate monitoring en route | 2(2.6%) | | Transfer time excessive | | 14(4.1%) | |

*EMS Emergency Medical Services; PFS paediatric flying squad*

*Modifiable Factor Impact: Major – factor which had clear negative impact on the outcome for the patient (worsened mortality or morbidity); directly and overwhelmingly important factor in the severity of illness/ death; Moderate – factor which on its own had minimal negative impact on the outcome but may have caused some morbidity and/ or extended the hospital/ PICU stay*
