# Supplementary material for: Pathways to Care for Critically Ill or Injured Children: A Cohort Study from First Presentation to Healthcare Services through to Admission to Intensive Care or Death
Source: PLoS One. 2016 Jan 5;11(1):e0145473. doi: 10.1371/journal.pone.0145473 (PMC4712128; doi:10.1371/journal.pone.0145473)
Supplement: S9 Table — (DOCX) [file pone.0145473.s010.docx]

**S9 Table. Compliance with Standards of Care for each facility and EMS level**

| **Facility/ EMS Level** | **N** | **TOTAL STDS APPLIED** | **% Critical Standards Met^a^** | **% Important Standards Met^a^** | **% Necessary Standards Met^a^** | **% Overall Met^a^** |
| --- | --- | --- | --- | --- | --- | --- |
| **General Practitioner** | 22 | 179 | 26.8 | 17.6 | 28.6 | 23.5 |
| **City Health Clinic** | 53 | 1016 | 49.8 | 57.7 | 41.3 | 50.9 |
| **CHC 24hr** | 106 | 2502 | 69.9 | 61.1 | 69.4 | 66.2 |
| **District Hospital** | 45 | 1090 | 77.5 | 72.7 | 73.3 | 74.4 |
| **Regional Hospital** | 50 | 1328 | 86.9 | 84.4 | 78.8 | 84.0 |
| **RCWMCH ED** | 239 | 5702 | 84.4 | 78.2 | 65.7 | 77.5 |
| **RCWMCH Ward** | 76 | 1452 | 89.8 | 81.4 | 67.1 | 79.6 |
| **OVERALL FACILITY** | **686** | **13572** | **79.0** | **72.4** | **65.6** | **73.1** |
| **EMS Primary (home/ scene)** | 65 | 1148 | 68.4 | 91.7 | 59.0 | 78.7 |
| **EMS Inter-facility** | 170 | 3179 | 72.9 | 93.6 | 63.2 | 81.0 |
| **EMS Paediatric Flying Squad** | 42 | 966 | 90.0 | 93.5 | 67.9 | 87.8 |
| **OVERALL EMS** | **277** | **5547** | **76.0** | **93.2** | **65.5** | **82.3** |

*Stds standards; CHC community health centre, RCWMCH ­ Red Cross War Memorial Children’s Hospital ED ­ emergency department; EMS emergency medical services (ambulance).*

*^a^ % met calculated by number of stds met/(stds met + stds not met)% i.e.% of all standards applied.*
