# Supplementary material for: Pathways to Care for Critically Ill or Injured Children: A Cohort Study from First Presentation to Healthcare Services through to Admission to Intensive Care or Death
Source: PLoS One. 2016 Jan 5;11(1):e0145473. doi: 10.1371/journal.pone.0145473 (PMC4712128; doi:10.1371/journal.pone.0145473)
Supplement: S10 Table — (DOCX) [file pone.0145473.s011.docx]

# **S10 Table. List of variables collected in Database.**

1. **Quantitative Data Collected For Each Case**

**Patient Demographics**

Date of Birth

Gender

PICU Admission Diagnosis

Medical/ trauma

Home suburb/ town

**Antenatal/ Developmental History**

Birth Weight (& binned)

Antenatal booking status

Mode delivery at birth

Gestational Age at Birth

Obstetric risk factors

Current weight

Current height

Current head circumference

**Social**

Mother’s status (alive/ dead/ sick)

Father’s status (alive/ dead/ sick)

Primary Caregiver (M/ F/ grandparent/ etc)

Where is child living

Nutrition (breast/ formula/ meals)

Nutritional Status

Feeding in first 6 months

**HIV/ AIDS Status**

Status (lab neg/ pos/ inf/ exposed)

PMCT given

ARV status child

ARV mother

ARV father

**Previous Medial History**

Immunization up to date?

Congenital anomaly? Details..

Convulsions?

Recent surgery?

Admission in last 3 months. (ICD 10)

Seen in PHC facility in last 3 months?

**Outcome**

Final status (alive/ died PICU/ death prior PICU)

Primary Diagnosis (ICD10)

Secondary Diagnoses (ICD10)

PIM2 Score

HIV status

HIV Clinical Stage

**Length of Stay**

RXH Admission Date

PICU Admission Date

PICU Discharge Date

Hospital Discharge Date or Death Date

Transferred to Ward (specified)

**Outcome**

Discharge Home?

30 day outcome. Details…

Records of referral/ follow up appointment

**Facility (for each facility or ward visited)**

Name of Facility/ Ward

Level of Facility

Medical/ trauma

Transferring Facility/ place. Details…

**Background**

Date illness started

Admission Diagnosis

Previous treatment. Details.

**Prior Consultations at this facility (in last month)**

Date

Diagnosis

Interventions

Follow up

**Arrival/ Admission**

Transport to facility

Arrival Date & time

For Operating Theatre only:

Start & End time anaesthetic/ surgery

Qualifications of first nurse assessing

Date/ time seen by nurse

Qualifications of first doctor assessing

Date/ time seen by doctor

Discussed/ seen by consultant?

**Initial Assessment**

Pulse Oximetry

Inspired Oxygen Concentration FiO_2_

Heart Rate

Blood Pressure

Respiratory rate

Temperature

Glasgow Coma Scale / AVPU

Haemo Glucose Test

Haemoglobin

Weight

Capillary Refill Time

SATS Triage Colour

**Interventions**

Airway

Respiratory Support

FiO2

Vascular Access

IV Fluids given/ type

Inotropes

Blood Transfused

FOR TRAUMA ONLY:

Intercostal Drain

Cervical Spine Protection

Spine Board

Splinting/ Backslab to fracture

Glucose given?

Drugs given:

Lab Investigations:

Urine test

Xray:

CT Scan… Time

**Progress/ Referral/ Discharge**

Admission overnight? Daily Details: Overall condition; Abnormalities; Issues

Critical Event Details…

Referal/ Discharge Details/ Date/Time

Transport out

Quality of Records

**EMS (for each transfer)**

Referring Facility

Receiving Facility

Background Diagnosis/ Prior Treatment

Date

EMS level (home/ scene/ flt/ PFS)

EMS type (primary/ secondary)

Med/ Trauma

Voucher Number/ Incident Number/ Vehicle Number

Ambulance Group

Priority Status

Paediatric Flying Squad Vehicle?

Incident date/ time

Dispatch date/ time

Arrival Scene date/ time

Departure date/ time

Arrival Destination date/ time

Crew Qualifications

**Initial Assessment**

Pulse Oximetry

Inspired Oxygen Concentration FiO_2_

Heart Rate

Blood Pressure

Respiratory rate

Temperature

Glasgow Coma Scale / AVPU

Haemo Glucose Test

Haemoglobin

Capillary Refill Time

Initial SATS Triage Colour

**Interventions**

Airway

Respiratory Support

FiO2

Vascular Access

IV Fluids given/ type

Inotropes

FOR TRAUMA ONLY:

Intercostal Drain

Cervical Spine Protection

Spine Board

Splinting/ Backslab to fracture

Glucose given?

Drugs given:

**EMS Disposal**

Handover by (qualification)

Handover to (qualification)

Records of Referral

1. **QUALITATIVE (INTERVIEW) DATA**

**General information**

Case Number

Injury Type

Date & time of interview

Length of interview (mins)

Type of Injury

Caregiver(s) Interviewed

Date of Birth

Address: Suburb

Address: Town

Address: Postcode

**Parent/ Caregiver Information**

Status of Mother

Age of Mother

Address (if different from child): Suburb

Address (if different from child): Town

Address (if different from child): Postcode

Marital Status

Language

Highest Educational Level

Employment Status

Status of Father

Age of Father

Address (if different from mother and child): Suburb

Address (if different from mother and child): Town

Address (if different from mother and child): Postcode

Highest Educational Level

Employment Status

Primary Caregiver

Status of Caregiver

Age of caregiver

Address (if different from mother and child)

Caregiver`s Marital Status

Highest Educational Level

Employment Status

Relation to child

**Household Information**

Monthly Household Income Bracket

Are you receiving any grant?

Type of grant

Is the father maintaining the child?

Is he paying Child Maintenance?

How many children are currently directly dependent on you (excluding the study child)?

Children in Family: Number of Male Siblings

Children in Family: Number of Female Siblings

Children in Family: Average Sibling Age

Children in Family: Sibling Age Range

Have any children died while under your care?

Please give details

Main source of energy for cooking

Main source of energy for heating

Main source of enery for lighting

Description of Area/Locality

Type of Dwelling

Construction of Dwelling Walls

Construction of Dwelling Roof

Number of rooms in dwelling

Water Source

Toilet

Assets owned: Radio

Assets owned: Television

Assets owned: Satellite dish

Assets owned: Refrigerator/freezer

Assets owned: Stove, gas or electric

Assets owned: Microwave oven

Assets owned: Washing machine

Assets owned: Motor vehicle

Assets owned: Motor cycle/ scooter

Assets owned: Bicycle

Assets owned: Canoe/boat

Assets owned: Cellular telephone

Assets owned: Computer

Assets owned: Internet service

**Health Facility Access**

Nearest Health Facility

Distance from your house (km)

Hours of Service at Facility

Mode of transport to nearest clinic

Cost of Single Trip (rands)

Is nearest 24hr service Health Facility different from above?

Nearest 24hr service Health Facility (if different from above)

Distance from your House (km)

Hours of Service at Facility

Mode of Transport to 24 hour Facility

Cost of Single Trip (rands)

If your child is very sick what do you do?

Specify ambulance number

If your child is injured badly what do you do?

Specify ambulance number

Why would you not call an ambulance in any case?

How long ago did these signs begin?

When did you decide to seek medical help?

When did you decide to seek medical help?: Interview Delay (hours)

When did you decide to seek medical help?: Pathway Delay (hours)

**Facility Information (for each facility visited)**

Name of Facility

Time you left home/previous facility

Mode of Transport

Cost of Single Trip (Rands Time of Arrival at Facility

Who directed you about where to go in the facility?

Vital signs taken soon after arrival?

Triage performed soon after arrival?

Understanding of triage system/pholosophy?

Was there a separate queue for children and adults?

Time of being attended by a health care provider

Were you (parent/carer) allowed to stay with the child throughout the treatment?

Were you satisfied?

Was your child in pain?

Were they given medicine for pain?

At what time?

Did you think they were relieved of pain following medication?

Was the child given OXYGEN?

When was it given?

Case of Gastro?

Was the child given any oral hydration?

Length of Oral Hydration (hours)

Was a drip put up?

When was the drip put up?

Where was the drip positioned?

Case of ASTHMA or Respiratory Distress?

Was the child nebulised?

When did the nebulisation occur?

Case of FEVER or SEPTIC SHOCK?

Was the child's urine tested?

Time of Urine Test

Case of Convulsions?

How frequent were the convulsions whilst you were still at home?

Do you feel that you were attended quite quickly?

And why do you say so?

Were you (parent/carer) allowed to stay with the child throughout the time?

Level of satisfaction

What was the outcome?

What were you told about your child's illness?

Who explained the child's illness to you?

In your opinion, could the medical care provided to your child have been improved?

How could the medical care be improved?

**EMS transfer (for each transfer)**

What time did the ambulance arrive to fetch child?

When did the ambulance arrive at destination facility?

How long did it take for the ambulance to come?

Did you think the ambulance crew seemed to have the right equipment?

Did you feel comfortable that your child was well treated?

Were you (parent/carer) allowed to stay with the child throughout the time?

Did you want to be there?

Did anything special/unusual happen on the ambulance trip in to hospital?

Please provide details

Did the ambulance crew explain to you what they were doing?

Did they comfort your child/reassure/explain (as appropriate for age)?

Is there anything you think could have made the ambulance transfer better?

Communication: Health Care Worker (HCW) spoke my language?

Communication Details

**Interview Conclusion Data**

Please tell me about anything you feel is important that was not covered in the interview

How stressful did you find taking part in this interview?

How did you find the length of the interview?

Would you be prepared to participate in this type of interview again?
